# A new argument against cooling by convective air eddies formed above sunlit zebra stripes

**DOI:** 10.1038/s41598-021-95105-4

**Published:** 2021-08-04

**Authors:** Ádám Pereszlényi, Dénes Száz, Imre M. Jánosi, Gábor Horváth

**Affiliations:** 1 Department of Biological Physics, ELTE Eötvös Loránd University, Pázmány sétány 1, 1117 Budapest, Hungary; 2 Deutsches Meeresmuseum, Katharinenberg 14-20, 18437 Stralsund, Germany; 3 Department of Physics, ELTE Eötvös Loránd University, BDPK, 9700 Szombathely, Hungary; 4 Department of Water and Environmental Policy, University of Public Service, Ludovika tér 1, 1083 Budapest, Hungary; 5 Max Planck Institute for Physics of Complex Systems, Nöthnitzer Strasse 38, 01187 Dresden, Germany

**Keywords:** Ecology, Physiology, Zoology, Optics and photonics, Physics

## Abstract

There is a long-lasting debate about the possible functions of zebra stripes. According to one hypothesis, periodical convective air eddies form over sunlit zebra stripes which cool the body. However, the formation of such eddies has not been experimentally studied. Using schlieren imaging in the laboratory, we found: downwelling air streams do not form above the white stripes of light-heated smooth or hairy striped surfaces. The influence of stripes on the air stream formation (facilitating upwelling streams and hindering horizontal stream drift) is negligible higher than 1–2 cm above the surface. In calm weather, upwelling air streams might form above sunlit zebra stripes, however they are blown off by the weakest wind, or even by the slowest movement of the zebra. These results forcefully contradict the thermoregulation hypothesis involving air eddies.

## Introduction

**Figure 1:**
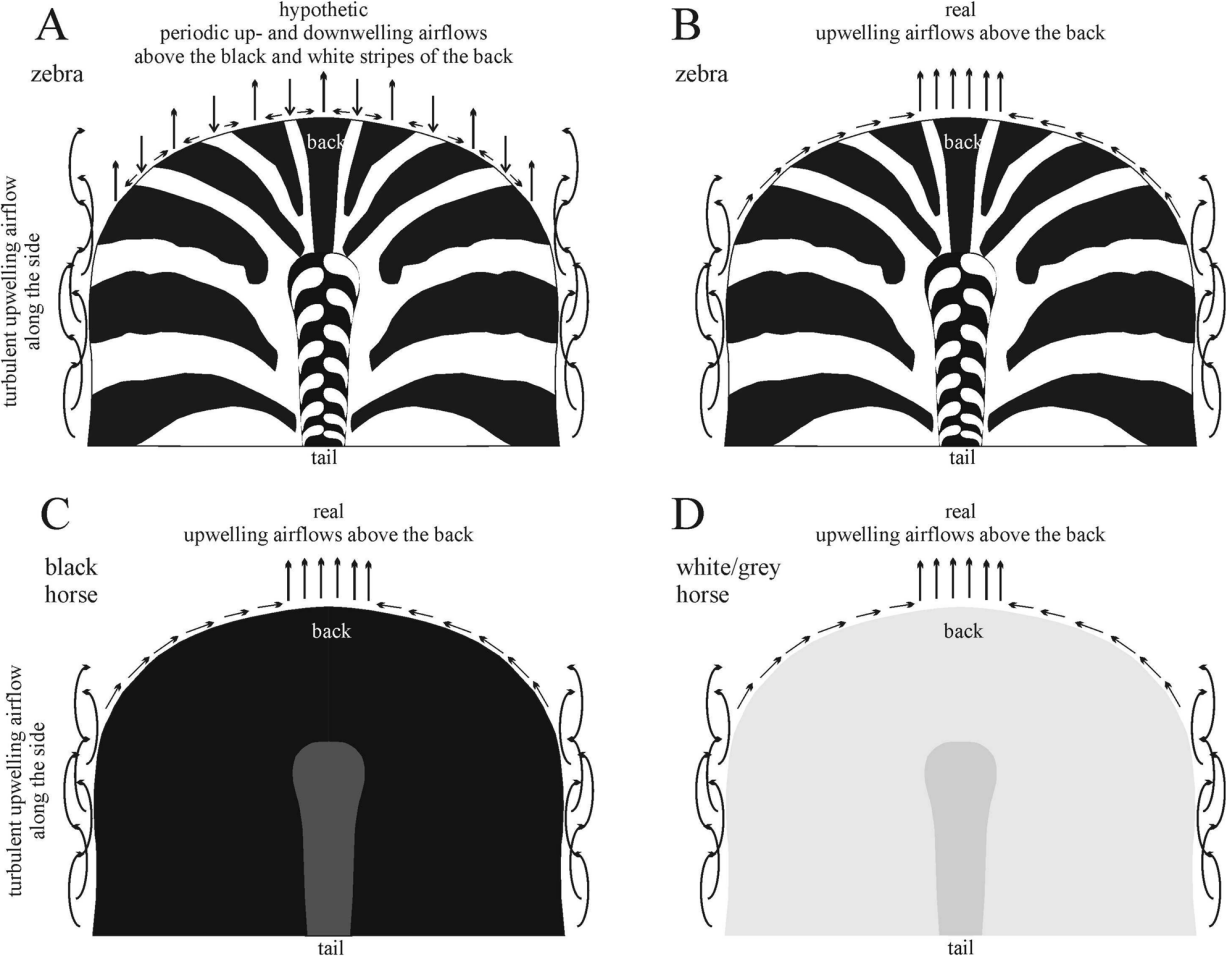
Schematic airflow above the sunlit body of zebras and horses under calm conditions. (**A**) Assumed convective eddies above the zebra back. According to the thermoregulation hypothesis, in sunshine upwelling/downwelling air streams form above the warmer black/cooler white zebra stripes which cool the body, while at the nearly vertical side surfaces the airflow is turbulent. (**B**–**D**) Real airflow conditions above the back of zebras (**B**), black horses (**C**) and white/grey horses (**D**) on the basis of schlieren imaging. (This figure was drawn by Gábor Horváth and Ádám Pereszlényi).

There is a long-standing debate about the possible functions of zebra stripes^1^. According to one hypothesis, a series of periodical convective air eddies form over the coat pattern of zebras under sunlit conditions and these eddies cool the body. Infrared photography of zebras showed that sunlit black stripes are warmer than sunlit white stripes and that the difference between them increases with rising air temperature, while at night temperature differences are reversed, with black stripes being cooler than white ones^2–5^. Due to their lower albedo (higher light absorbance), sunlit dark stripes are warmer than neighbouring white stripes^6^, thus upwelling warmer air streams may form above the darker areas which are expected to be replaced by the downwelling cooler air from above the adjacent white stripes (Fig. 1A). In principle, cooling could be driven by these assumed convective eddies due to transporting warm air away over the warmer black stripes. Furthermore, convection might accelerate the evaporation of sweat on the zebra skin^7^. There are roughly two opposing opinions in the literature on the hypothesis of thermoregulation.

On the one hand, a few sceptic authors presented theoretical arguments and/or experimental data which do not support the assumed cooling effect by zebra stripes: Infrared camera measurements of Caro^1^ did not confirm lower surface temperatures of sunlit zebras compared to sympatric sunlit ungulates (giraffe, impala and buffalo). Caro^1^ has emphasized that an almost ever-blowing wind in the field can easily disrupt convective eddies above sunlit zebras, even if they form at all. In addition, if in calm weather such air eddies formed above stationary zebras, they would quickly break off when the animals move during grazing or running. Horváth et al.^5^ presented experimental evidence against cooling by zebra stripes. They performed field experiments and thermographic measurements to study whether thermoregulation might work for zebra-striped bodies. The zebra body was modelled using water-filled metal barrels covered with horse, cattle and zebra hides which were homogenous black, white, grey or striped. The barrels were installed in the open air for four months while their core temperatures were measured continuously. There were no significant core temperature differences between striped and grey barrels, even on many hot days, independently of the air temperature and wind speed. The average core temperature of the barrels increased from being covered by white cattle through grey cattle, real zebra, artificial zebra, grey horse to black cattle skins. These field experiments demonstrated that zebra-striped coats do not keep the body cooler than grey coats.

On the other hand, earlier authors had the opposite argument^6,8–11^, but only a few of them have presented data to support it. Larison et al.^12^ matched environmental variables to striping intensity from populations of plains zebra to support the cooling idea. More recently, Cobb and Cobb^7^ measured the temperatures of black and white stripes on two living zebras and a zebra hide, throughout separate sunny days in Kenya. They registered 12–15 °C differences between the temperatures of black and white stripes of living zebras in daylight, while this difference reached 16 °C on zebra hides. In their opinion, the surfactant equid protein, called latherin, could decrease the surface tension of zebra sweat, which might facilitate evaporative cooling at the hair tips. Although the original hypothesis assumed cooling by periodic convective eddies, they suggested that the sharp temperature difference between the black and white stripes could cause chaotic air movement above the hair surface, thus enhancing evaporative heat dissipation. They also proposed that this cooling mechanism could explain the lower temperatures of the stripes of living zebras than those of the inanimate hide. They observed that the hair of black zebra stripes can be erected separately from white stripes, which could further refine the mechanism of sweat evaporation. According to Cobb and Cobb^7^, their data and observations suggest that the primary function of zebra stripes may be thermoregulation (cooling).

However, simple measurements of the diurnal change of the temperature difference between adjacent sunlit white and black zebra stripes cannot answer the question whether sunlit zebra stripes can or cannot promote convective air eddies. The assumption of cooling by zebra stripes should be studied by more appropriate experimental methods. The formation of convective air eddies above sunlit stripes should be experimentally studied by schlieren imagery for a number of temperature differences between adjacent white and black stripes, and wind speeds to mimic the zebra body and the circumstances in which it lives. This is the aim of the present study. Only such an experiment can provide evidence or counter-evidence for the alleged cooling effect of zebra stripes. Here, we first describe the thermographic and schlieren imaging methods with which numerous light-heated test surfaces were investigated in the laboratory. Then, we present our results obtained with evaluation of schlieren video sequences. After discussing the observations, we finally draw conclusions that refute the hypothesis of the formation of periodical convective air eddies above sunlit zebra stripes.

## Materials and methods

### Test surfaces

We used smooth and hairy, homogeneous and striped (with different widths of black and white stripes) test surfaces. In order to mimic the curved zebra back, all test surfaces had a convex cylindrical shape (length: 15 cm, radius: 7 cm). In the schlieren measurements (see subsection “Schlieren imaging”), the cylinder’s horizontal long axis was perpendicular (Supplementary Fig. S1A,B) or parallel (Supplementary Fig. S1C) to the collimated horizontal light beam illuminating the target area, while the stripes were perpendicular (Supplementary Fig. S1A) or parallel (Supplementary Fig. S1B) to the cylinder’s long axis. Supplementary Tables S1 and S2 list the patterns, colours and names of the 8 smooth and 10 hairy test surfaces. The smooth surfaces were composed of cardboard squares (15 cm × 15 cm) (Supplementary Fig. S2). The hairy surfaces were composed of cattle, horse and zebra hides glued by dextrin to a cylindrical (length: 15 cm, radius: 7 cm) gypsum base (Supplementary Fig. S3). Surfaces hsc1(7b7w)perp, hsc1(8b7w)par, hsc3(3b2w)perp and hsgc3(3s2L)perp were used to model that the black stripes of zebras could be separately erected, while the white remained flat^7^. No animals were killed, the horse and cattle hides were provided by Hungarian horse and cattle keepers, while the zebra hide was obtained from a Hungarian zoological garden.

### Thermography of lamplit test surfaces

Using a thermocamera (VarioCAM, Jenoptik Laser Optik Systeme GmbH, Jena, Germany, nominal precision of ± 1.5 K, with relative pixel-to-pixel precision < 30 mK) we measured the temperature distribution of all test surfaces under the same circumstances as occurred during the schlieren measurements. The validation and calibration of our thermocamera with a contact thermometer (GAO Digital Multitester EM392B 06554H, EverFlourish Europe GmbH, Friedrichsthal, Germany, nominal precision of ± 1˚C) were described in the Electronic Supporting Material of Horváth et al.^13^.

Along a horizontal straight line we calculated the followings: average *T*_ave_ ± standard deviation σ_T_ of the surface temperature *T*, minimum *T*_min_ and maximum *T*_max_ of *T*, average Δ*T* ± standard deviation σ_ΔT_ of the temperature difference between the adjacent local minima and maxima of *T*.

### Schlieren imaging

**Figure 2:**
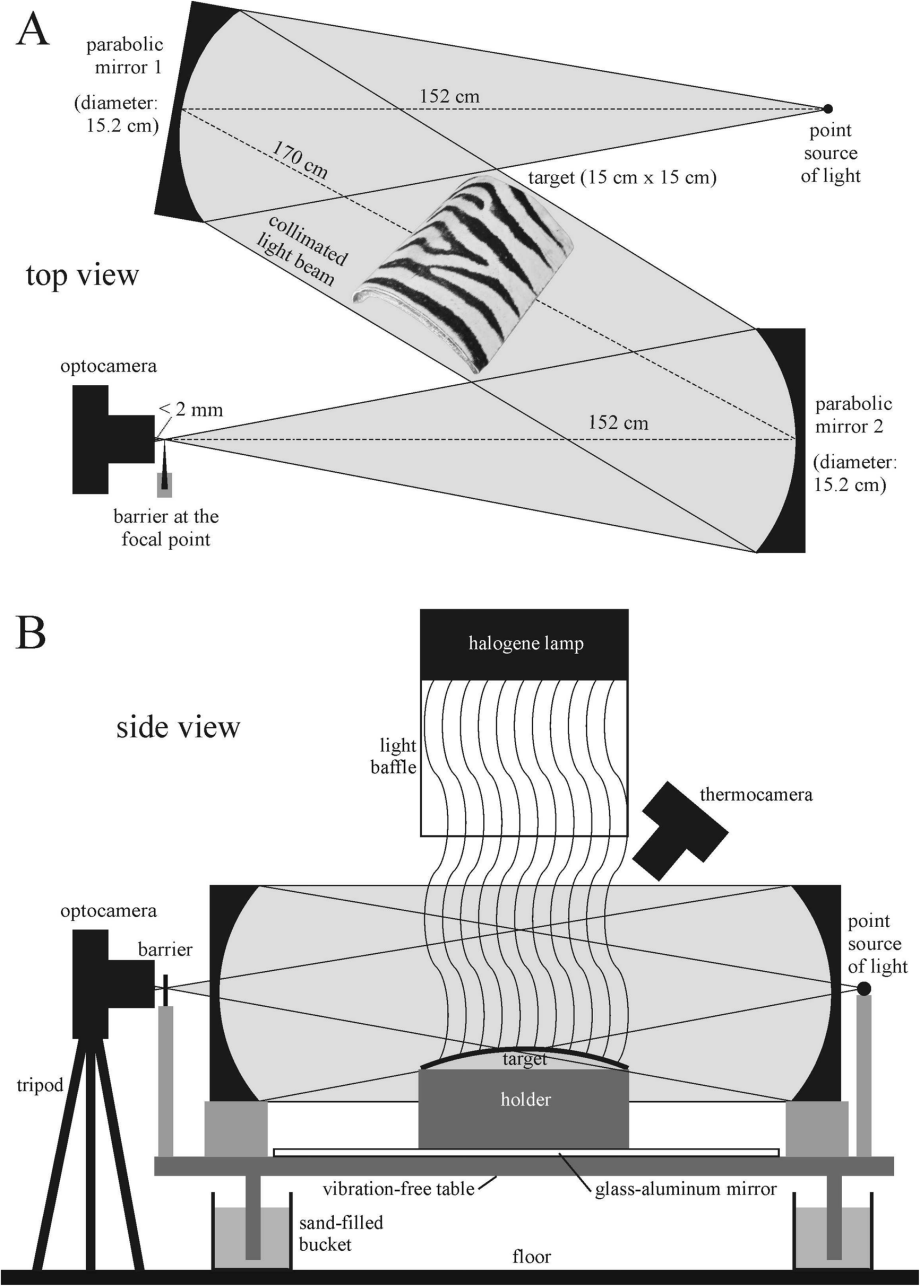
Not-to-scale schematic top (**A**) and side (**B**) views of the schlieren imaging device used to visualize the spatiotemporally changing air streams above light-heated test surfaces.

We used schlieren imaging to study the airflow above lamplit test surfaces. The experimental setup of our schlieren device is schematically shown in Fig. 2. With this optical technique the spatiotemporal change of the temperature-dependent refractive index of air can be visualized^14^. In the schlieren images, we looked for nearly vertical dark-bright stripe pairs above the lamplit test surfaces, because these stripes visualize the air streams that could be the assumed convective eddies above sunlit zebra stripes possessing a cooling effect. Our schlieren setup was composed of two parabolic mirrors (Edmund Optics, Barrington, NJ, USA), both with a diameter of 15.2 cm and focal length of 152 cm (Fig. 2 and see also Supplementary Figs. S4 of Horváth et al.^13^). The lamplit test surfaces were placed into the horizontal collimated light beam of the schlieren equipment, the light source of which was composed of a white LED light (3 Watt, 4000 K). We recorded the schlieren images/videos with a Nikon D5600 DSLR camera used within 120 mm focal length in Full HD resolution (1920 × 1080 pixels). The blade in front of the lens was vertical, thus the horizontal component of the refraction became visible. To model the heating of the test surfaces by sunlight, we used a 400 W, 3500 K halogen lamp producing intense white light corresponding to sunlight (Everflourish Hungary Ltd., Budapest, Hungary) placed vertically above the target at a height of 60 cm. Below the lamp there was a vertical tube (length: 43 cm) with a square cross-section (19 cm × 14.5 cm), the inner walls of which were covered with aluminum foil to reflect the lamplight. The bottom of this tube was 17 cm above the target (test surface). Under the target area there was a horizontal plane mirror (100 cm × 70 cm) and a T-shaped stand covered with aluminum foil which held the test surface at the appropriate height. The function of the plane mirror and the aluminum covering of the stand was to minimize the warming up of the surrounding area.

After placing the test surface onto the T-holder in the target area, we waited 5 min to heat up the test surface by lamplight to a constant temperature. 5 min proved to be enough for the stabilization of the target’s temperature. Schlieren sequences were recorded when (i) the long axis of the cylindrical test surface was perpendicular to the camera’s optical axis (3 min, Supplementary Fig. S1A, B), (ii) the long axis was parallel to the optical axis (3 min, Supplementary Fig. S1C), and (iii) the test surface was removed from the T-holder in order to record the background (1 min).

### Evaluation of schlieren images and video sequences

As a result of schlieren imaging, we got a video sequence of the air layer above a given lamplit test surface placed on the T-holder. We converted this video sequence to single png images cropped to 908 × 908 pixels (Supplementary Fig. S4A) and made them grey-scaled. Averaging the intensity value—ranging from 0 (black) to 255 (white)—of every pixel of the images of the 1-min-long sequence taken above the same lamplit air layer when the test surface was removed from the T-holder, we got the averaged schlieren image of the background (Supplementary Fig. S4B). Then, we composed the difference of the background image and the original image resulting in a filtered schlieren image (Supplementary Fig. S4C), the pixel intensity of which was calculated from the formula *I*_filtered_ = 125—(*I*_background_—*I*_original_), where *I*_background_ is the pixel intensity of the averaged background image (without the test surface) and *I*_original_ is the pixel intensity of the grey-scaled image with the test surface. This filtering was necessary to ensure a homogeneous middle grey (with a constant pixel intensity *I*_grey_ = 125) shade of the background. Note that if *I*_background_ = *I*_original_, then *I*_filtered_ = 125.

To characterize the spatiotemporal change of pixel intensity induced by different air streams above a given lamplit test surface, we selected an upper (7 cm above the surface) and a lower (0.5 cm above the surface) horizontally elongated rectangular window (smooth surfaces: width of upper and lower windows was 612 pixels = 10.5 cm, hairy surfaces: width of upper and lower windows was 612 pixels and 698 pixels = 12 cm, respectively, height of both windows was 40 pixels = 0.7 cm above both surface types) in the filtered image (Supplementary Fig. S4D). Within both rectangles, we averaged the pixel intensity *I* for the vertical pixel column (40 pixels) along the horizontal axis *x*. The resulting *I*(*x*) curve was smoothed by a Gaussian filter with standard deviation of σ = 5. Then, we found the local minima of the Gaussian-smoothed curve *I*(*x*), which are shown by vertical lines in the top and bottom of the plots. The sites of these local minima of *I*(*x*) coincide with those of the bright-light stripe pairs of the filtered schlieren image visualizing air streams with gradient indices of refraction induced by local temperature gradients.

To characterize an *I*(*x*) curve, we counted the number *N*_min_ of local minima of *I*(*x*), the difference Δ*I* = *I*_max_—*I*_min_ along this curve, and the average displacement *d*_ave_ of each local minimum. *d*_ave_ was calculated as follows: for the *i*-th local minimum, we found the closest local minimum in the frame recoded 1 s later, calculated the displacement *d*_i_ between these minima and averaged them as ${d_{{{\text{ave}}}} = \sum {d_{i} } /N_{{{\text{min}}}} }$. If there was no or only one local minimum, then *d*_ave_ = 612 (= pixel width of the selected window).

To analyze the behaviour of air streams above the lamplit test surfaces, we identified each local minimum of *I*(*x*) and characterized its movement with its lifespan *t* (from appearance to disappearance, e.g. *t* = 3 × 33 ms corresponds to 3 frames), covered distance *d*_covered_ (e.g. if the movement was 5 pixels to left between the 1st and 2nd frames, and the movement was 10 pixels to right between the 2nd and 3rd frames, then *d*_covered_ = 5 + 10 = 15 pixels), mean speed *v*_mean_ = *d*_covered_/*t*, maximum distance *d*_max_ = *x*_max_—*x*_min_, and start–end distance *d*_se_ =|*x*_start_—*x*_end_| between its start (*x*_start_) and end (*x*_end_) points. For statistical analyses, we used only local minima with lifespan *t* > 1 s. Since in the upper rectangular window of the filtered schlieren images the lifespan *t* of local minima of *I*(*x*) was very short, we performed statistics only for the lower rectangular window.

For the statistical analysis of the characteristics of *I*(*x*) (*N*_min_, Δ*I*, *d*_ave_) and air stream behaviour (*t*, *d*_covered_, *v*, *d*_max_, *d*_se_), we applied Principal Component Analysis (PCA) and fitted ellipses to component scores with 95% confidence interval. We applied Wilcoxon rank sum test with Bonferroni correction for the datasets. Because of the large sample size of the dataset *N*_min_, Δ*I*, *d*_ave_—for smooth test surfaces 4475 observations per surface and for hairy test surfaces 5369 observations per surface—, the Wilcoxon rank sum test would result in highly significant differences for almost all comparisons^15^. Therefore, we used a Monte Carlo approach, where we randomly selected 250 (≈ 5% of the full dataset) samples, ran the Wilcoxon rank sum test with Bonferroni correction, recorded the results, repeated this 499 times (to have 500 runs) and finally the average of the results of the 500 runs was calculated. The data evaluation was made by our custom-written scripts in Python programming language. For statistical analyses we used the R statistical package 3.6.3^16^.

### Disturbance caused by an artificial wind and a butterfly

To demonstrate the influence of very weak winds on the air streams above lamplit test surfaces, we blew air by a compressor with a press of 1.6 bar from 1.5 m above the following test surfaces: ss1(8b7w), hsz(8b7w)perp, hsh1(8b8w)perp and hhbc. The compressor tube ended in an air-blow gun. Before the measurements, the air pressure was 4 bar in the 3-litre compressor tank, and the regulator was set to 1.6 bar. The compressor was turned off prior to recording a given sequence and during the measurement the air-blow gun was used to generate horizontal “wind” until the pressure decreased to 1 bar (atmospheric pressure). The artificial wind speed created by the compressor was measured (with accuracy < 0.1 km/h) with the same meteorological station (Conrad Electronic, equipment no: 672861) which was used in the field experiments of Horváth et al.^5^.

To demonstrate the instability of vertical air streams above the test surfaces, we recorded the trail/trace of a butterfly model in schlieren images above the following test surfaces: ss1(8b7w), hsz(8b7w)perp, hsh1(8b8w)perp and hhbc. The artificial cardboard butterfly (wingspan: 4.5 cm) hung from a thread (50 cm) weighted with an M6 size brass nut. This butterfly model was moved by the thread (swung left to right or shake up and down) above the illuminated surfaces and airflow patterns were recorded. The aim of this was to demonstrate that the tiny air disturbances caused by a butterfly can disrupt the upwelling air streams above sunlit zebra stripes.

### Typical wind speed in the field

To demonstrate the typical range of wind speed in the open air in the summer months, we used the wind speeds recorded during the field experiments of Horváth et al.^5^ with an automatic meteorological station (Conrad Electronic, equipment no: 672861). This station was installed from 10 June to 19 September 2017 in a horse farm in Göd (47°43′N, 19°09′E, northern Hungary), where it measured the air temperature *T*_air_ and wind speed *w* continuously at a height of 1 m above the ground. Further details about the setup of this station can be read elsewhere^5^. We used Spearman rho test to find correlation between the measured wind speeds and air temperatures.

### Ethical approval and informed consent

For our studies no permission, licence or approval was necessary. We confirm that no animals were killed specifically for the purpose of this study.

## Results

### Thermograms of lamplit test surfaces

**Figure 3:**
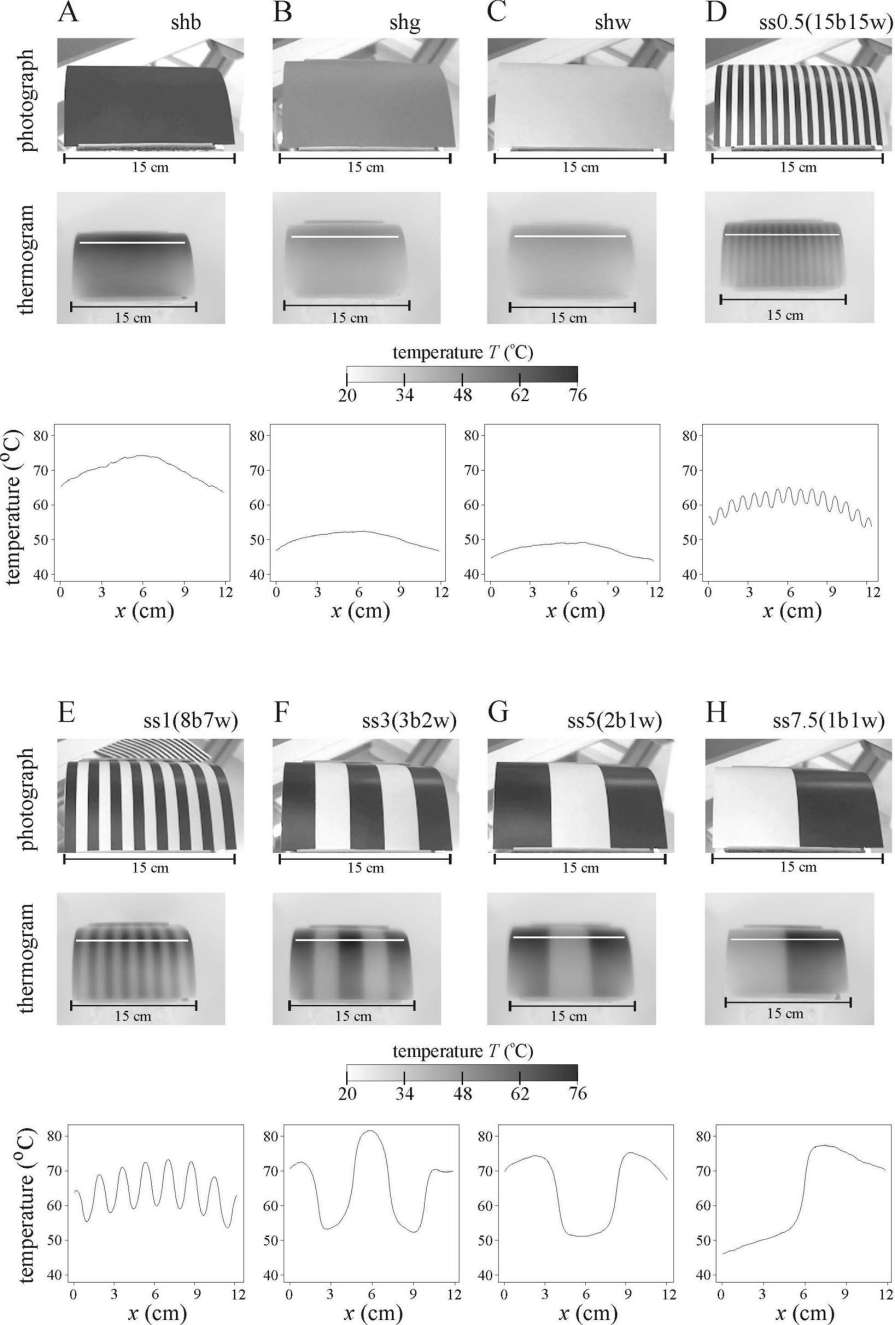
Thermography of the eight lamplit smooth horizontal cylindrical striped test surfaces (**A**) shb, (**B**) shg, (**C**) shw, (**D**) ss0.5(15b15w), (**E**) ss1(8b7w), (**F**) ss3(3b2w), (**G**) ss5(2b1w) and (**H**) ss7.5(1b1w) used in the schlieren experiments. Photographs, thermograms and the change of surface temperature *T* (°C) along the horizontal white line shown in the thermograms. The start and end points of these lines correspond to the first and last data points of the *T*-curves, respectively.

**Figure 4:**
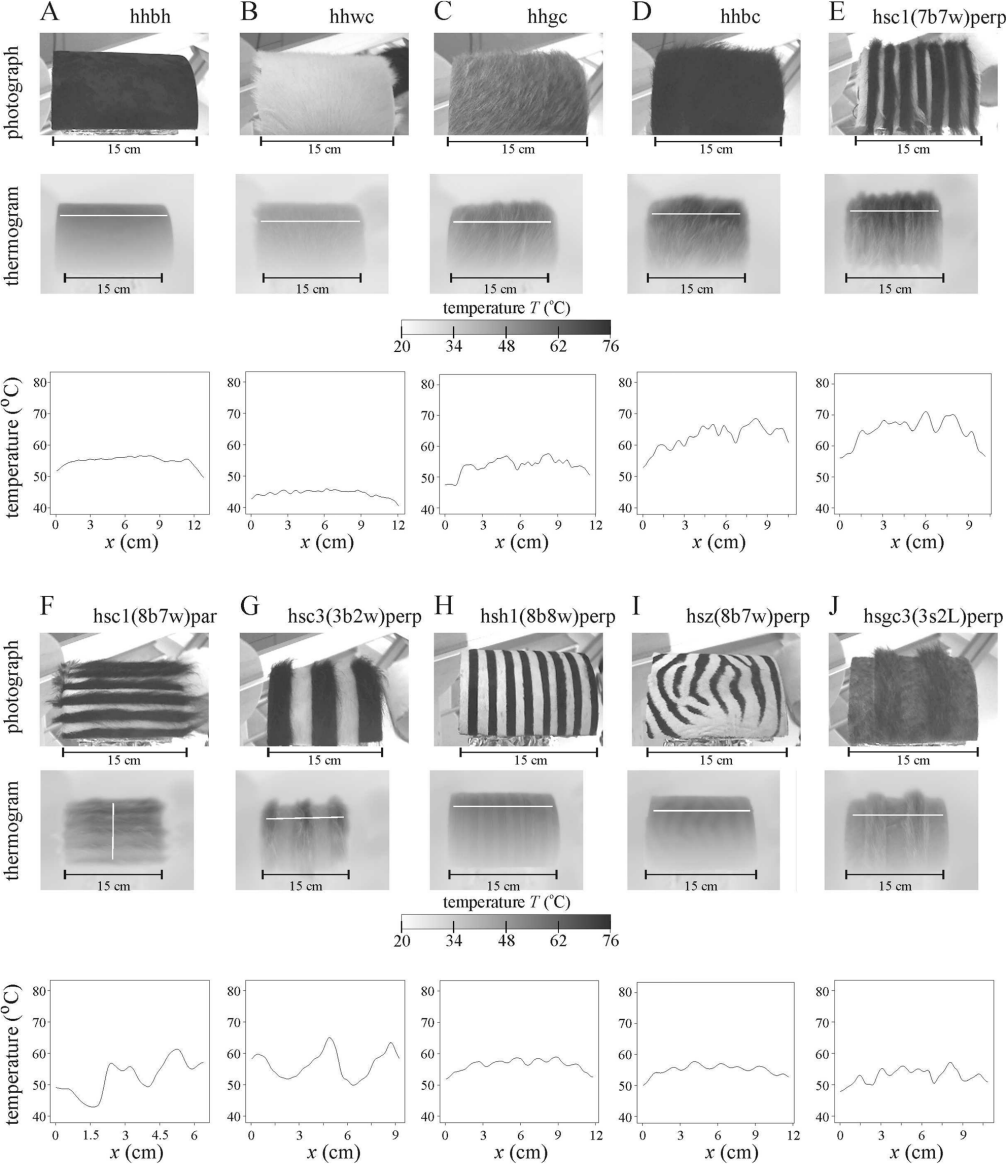
Thermography of the ten lamplit hairy horizontal cylindrical striped test surfaces (**A**) hhbh, (**B**) hhwc, (**C**) hhgc, (**D**) hhbc, (**E**) hsc1(7b7w)perp, (**F**) hsc1(8b7w)par, (**G**) hsc3(3b2w)perp, (**H**) hsh1(8b8w)perp, (**I**) hsz(8b7w)perp and (**J**) hsgc3(3s2L)perp used in the schlieren experiments. Photographs, thermograms and the change of surface temperature *T* (°C) along the horizontal or vertical white line shown in the thermograms. The start and end points of these lines correspond to the first and last data points of the *T*-curves, respectively.

On the thermograms of the smooth and hairy test surfaces we observed that the wider the stripes were, the higher was the surface temperature difference Δ*T* between the black and white stripes (Figs. 3, 4, Supplementary Tables S3, S4). Furthermore, the thermograms of Figs. 3 and 4 demonstrated that in our present laboratory schlieren experiments, the temperature distributions of our light-heated test surfaces were similar to those of sunlit living horses and zebras measured in earlier field experiments^5,13,17^. Thus, the thermal characteristics of our lamplit test surfaces studied in the laboratory with schlieren imaging modelled well the real thermal features of sunlit horses and zebras under natural field conditions.

### Schlieren images of air streams above smooth test surfaces

**Figure 5:**
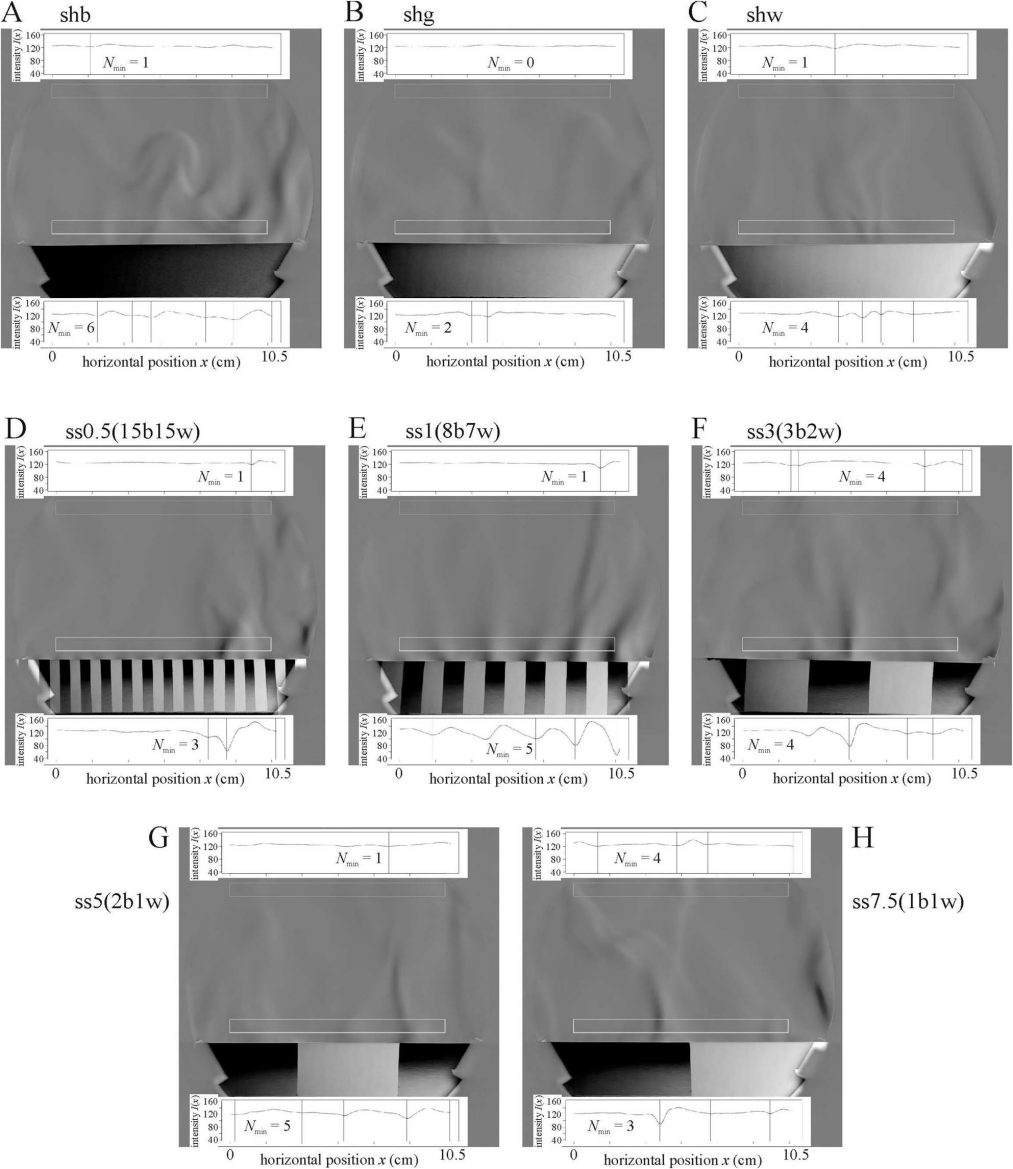
Schlieren images of the lamplit cylindrical black shb (**A**), grey shg (**B**), white shw (**C**) and striped ss0.5(15b15w) (**D**), ss1(8b7w) (**E**), ss3(3b2w) (**F**), ss5(2b1w) (**G**), ss7.5(1b1w) (**H**) smooth test surfaces when the curved stripes were parallel and the cylinder’s horizontal long axis (corresponding to the head-to-tail direction on the back of an ungulate) was perpendicular to the light beam. The upper and lower horizontally elongated rectangular windows are shown with a bright perimeter. The top and down insets are the plots of the averaged pixel intensity *I*(*x*) (vertical axis) along the horizontal axis *x* of the rectangular windows. The number *N*_min_ of local minima of *I*(*x*) depicted with vertical lines is given in the plots. Directly above a given monocoloured surface region a constant grey horizontal bar appears, the greyness of which depends on that of the surface. This bar is due to the thin, non-steaming air layer stuck to the surface.

Upwelling warm air streams emerged from the lamplit warm smooth test surfaces (see Fig. 5 and Supplementary Video Clip VC1). In the schlieren images, the greyness differences demonstrate the temperature difference between these warmer air streams and the colder surrounding air. The brighter, and thus the colder the lamplit test surface is, the weaker is this deflection of light passing through the air above the test surface (Fig. 5).

**Figure 6:**
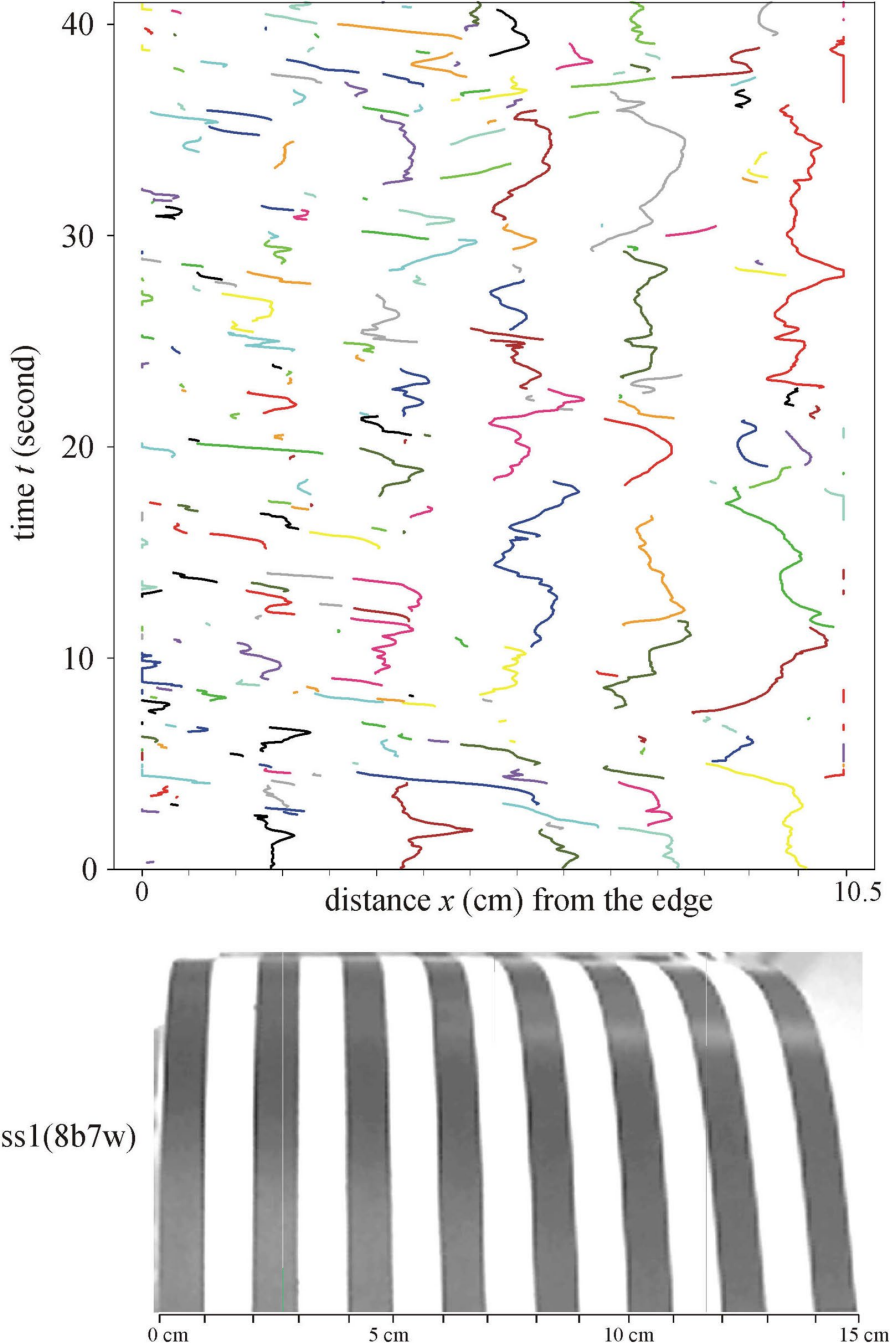
Horizontal movement of the upwelling air streams with individual colours identified in the lower window of the schlieren image above test surface ss1(8b7w). The curved stripes of the cylindrical test surface were parallel and the cylinder’s horizontal long axis was perpendicular to the light beam. Vertical axis: time *t* (second). Horizontal axis: horizontal position *x* (cm) of streams. This figure shows the first 40 s corresponding to the first 1000 frames. The stream colours are the same as the colours of vertical lines depicting the local minima of the averaged pixel intensity *I*(*x*) in the lower window of Supplementary Video Clip VC1.

**Figure 7:**
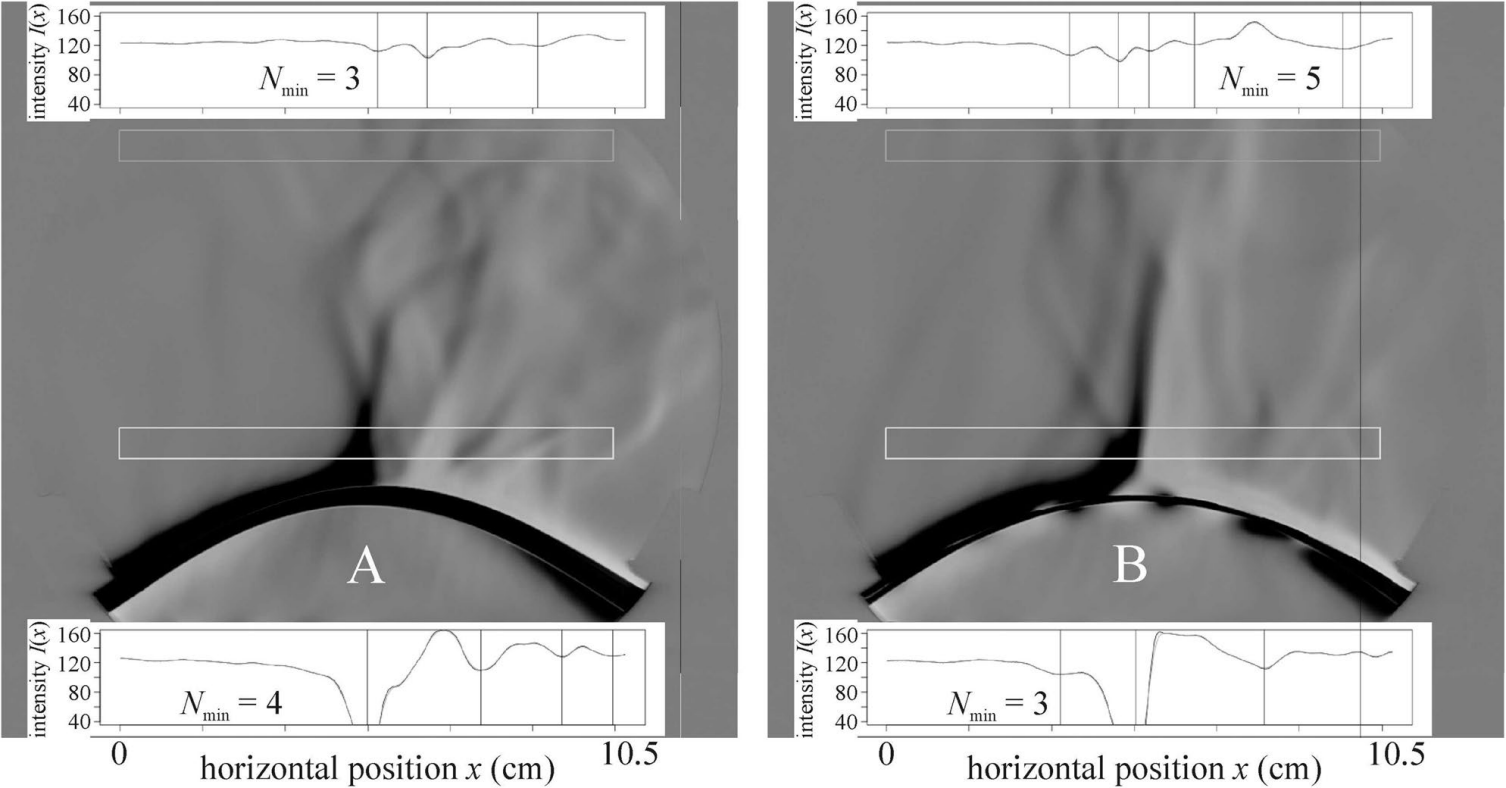
Schlieren images of the lamplit cylindrical striped smooth test surface ss1(8b7w) when the cylinder’s horizontal long axis was parallel to the light beam, and the curved stripes were perpendicular (**A**) or parallel (**B**) to the long axis (see also Supplementary Video Clips VC2 and VC3). Other conventions are the same as in Fig. 5.

Above all test surfaces the warmed-up air always flows upwards and many upwelling air streams drift more or less horizontally (see Fig. 6). The upward flowing warm air layer sticks to the curved striped surface, follows its curvature, breaks away from the uppermost line of the cylindrical surface and rises upwards (see Fig. 7 and Supplementary Video Clips VC2, VC3). This is true for all striped test surfaces, independently of the orientation (parallel or perpendicular to the long axis of the cylindrical substrate) and type (smooth or hairy) of the surface. When the curvature of the surface was parallel to the light beam, the upwelling airflow was fed laterally. These findings disprove the original hypothesis (Fig. 1A) that a series of air eddies composed of upwelling warm (above warmer black stripes) and downwelling cool (above colder white stripes) air streams form above sunlit zebra stripes. The real airflow above the zebra and non-striped ungulates is shown in Fig. 1B–D.

The statistical analyses showed that test surface ss1(8b7w) with 1 cm stripe width is statistically different for all three variables *N*_min_(lower), *d*_ave_(lower) and Δ*I*(lower) = *I*_max_—*I*_min_ measured in the lower window compared to other striped surfaces (Supplementary Table S5). However, considering these three variables in the upper window, there was no significant difference among the striped surfaces, except ss3(3b2w) versus ss5(2b1w) for *N*_min_(upper) with *p* = 0.0358, and *d*_ave_(upper) with *p* = 0.0152 variables. This suggests that though the 1 cm stripe width has an effect for the air stream formation, this effect can be observed only in the proximity of the surface, while in the upper window (7 cm above the surface) this effect practically does not occur (Supplementary Fig. S5). One could have expected that the homogeneous grey surface shg would be more similar to the striped surfaces (since the averaged intensity is equal). Despite this, surface shg is similar to the homogeneous white surface shw (Supplementary Fig. S5), and significantly differs only in variables *N*_min_(lower) (*p* = 0.0307) and Δ*I*(lower) (*p* = 0.00523, Supplementary Table S5). This can be explained with the fact that the lamplit black stripes are warmer than the grey surface, therefore the formation of upwelling air streams is more intense above the black-white striped surfaces. This explanation is supported by our finding that the homogeneous black surface shb is similar to the striped ones considering all variables, and the difference between the grey and striped surfaces is higher than that between the black and striped ones (Supplementary Fig. S5, Supplementary Table S5). For more detailed analysis see Supplementary Results.

The statistical analysis of air stream behaviour above the smooth test surfaces (see Supplementary Results) showed that the most stable upwelling air streams formed above test surface ss1(8b7w). This finding supports our observation that the stripe borders catalyze the stream formation and this effect is the strongest above ss1(8b7w).

### Schlieren images of air streams above hairy test surfaces

**Figure 8:**
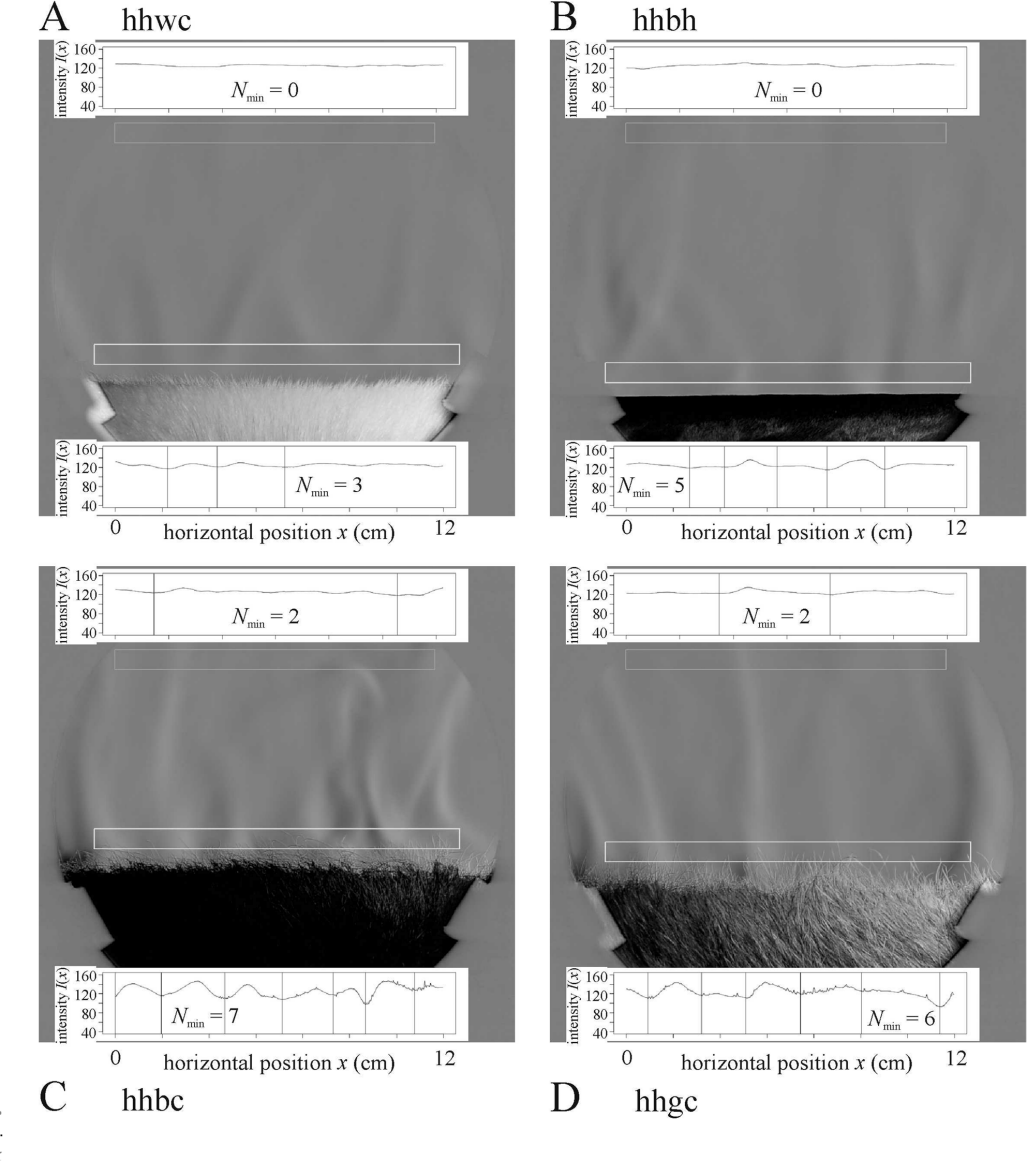
Schlieren images of the lamplit cylindrical homogeneous hairy test surfaces when the cylinder’s horizontal long axis was perpendicular to the light beam. (**A**) long-haired white cattle hide hhwc, (**B**) short-haired dark brown horse hide hhbh, (**C**) long-haired black cattle hide hhbc, (**D**) long-haired grey cattle hide hhgc. Other conventions are the same as in Fig. 5.

**Figure 9:**
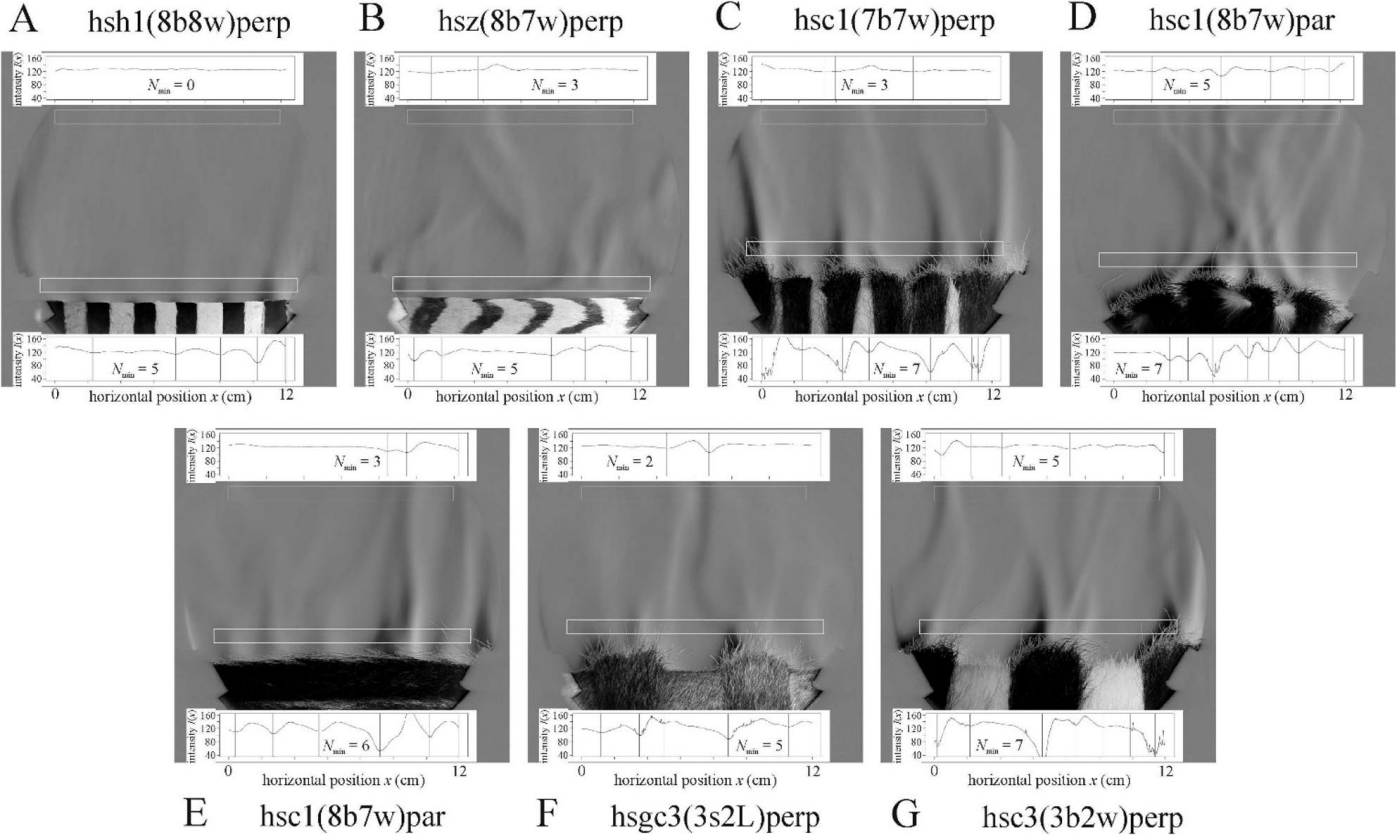
Schlieren images of the lamplit cylindrical striped hairy test surfaces when the curved stripes were parallel and the cylinder’s horizontal long axis was perpendicular to the light beam. (**A**) hsh1(8b8w)perp composed of short-haired dark brown and white horse hide stripes of 1 cm width. (**B**) short-haired zebra hide hsz(8b7w)perp (average white stripe width: 2 cm, average black stripe width: 1 cm). (**C**) hsc1(7b7w)perp composed of long-haired black and short-haired white cattle hide stripes with 1 cm width. (**D**) hsc1(8b7w)par composed of long-haired black and short-haired white cattle hide stripes with 1 cm width. (**E**) hsc1(8b7w)par composed of long-haired black and short-haired white cattle hide stripes with 1 cm width. (**F**) hsgc3(3s2L)perp composed of long-haired black and short-haired grey cattle hide stripes with 3 cm width. (**G**) hsc3(3b2w)perp composed of long-haired black and short-haired white cattle hide stripes with 3 cm width. Other conventions are the same as in Fig. 5.

Above the short-haired test surfaces composed of white cattle (Fig. 8A), horse (Figs. 8B, 9A) and zebra (Fig. 9B) hides the air stream behaviour was similar to that observed above the smooth test surfaces. However, above the long-haired test surfaces composed of black and grey cattle hides (Figs. 8C,D, 9C–G), the long fur helped the stream formation, as happened at the stripe borders of striped smooth surfaces. The long fur provides small peaks which help the upwelling warm air stream to secede. Furthermore, the long fur reduced the horizontal drift of streams more effectively than the stripe borders. When the hairy horizontal stripes and the cylinder’s horizontal long axis were parallel to the light beam (Fig. 9D), the warm air flowed upward from the lower sides to the uppermost horizontal line of the cylinder mantle, as above smooth surfaces (Fig. 7A,B). Above hairy test surfaces consisting of long- and short-haired curved stripes parallel to the light beam (Fig. 9C,G,F), the long-haired stripes acted as a curved surface parallel to the light beam, and thus upwelling air streams always formed above the long-haired stripes.

According to the statistical analysis of hairy test surfaces, in the lower window the most upwelling air streams formed above the homogeneous black surface hhbc which differed highly significantly from all other hides (Supplementary Fig. S9, Supplementary Table S9). The results suggests that the long hairs catalyze the formation of upwelling warm air streams even more than the stripe borders. The upwelling air streams were more stable above the long-haired surfaces than above the short-haired ones (see Supplementary Results).

Considering any variable, there was no statistical difference between the two orientations (long axis perpendicular or parallel to the light beam) of surface hsc1(8b7w)par. However, when the cylinder’s horizontal long axis was parallel to the light beam, most of the streams moved to the cylinder’s uppermost (middle) line, while when the cylinder’s long axis was perpendicular to the light beam, such a tendency towards the middle did not occur.

### Disturbances caused by an artificial wind and a butterfly

**Figure 10:**
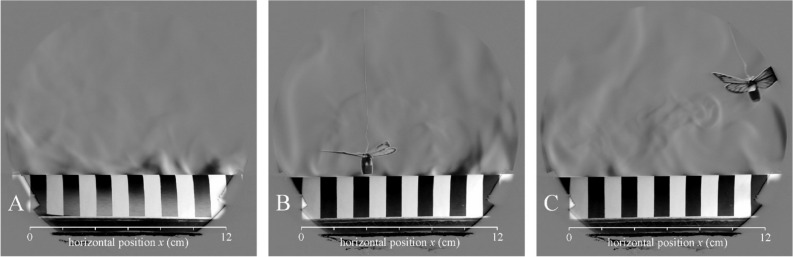
Schlieren images of the lamplit cylindrical striped smooth test surface ss1(8b7w) with 1 cm stripe width when the curved stripes were parallel and the cylinder’s horizontal long axis was perpendicular to the light beam. (**A**) A very weak wind was blown above the test surface. (**B**) A butterfly model flew up and down above the test surface in calmness. (**C**) A butterfly model flew from left to right above the test surface in calmness.

During our demonstrative wind experiments, the compressor regulator was set to 1.6 bar. Using the windmill (wind speed sensor) of our portable meteorological station, we tried to measure the wind speed generated above the striped surfaces by the air-blow gun at a distance of 1.5 m. However, the generated wind was so weak that it could not rotate the windmill, thus the nominal wind speed was 0 km/h. Even this very weak wind was enough to blow and disrupt the formation of air eddies above the striped test surfaces (see Fig. 10A and Supplementary Video Clips VC4-VC7).

In the butterfly experiment we demonstrated the high instability of upwelling air streams above the test surfaces. Even the movement of a small artificial butterfly can distract these air streams, which, however, reform immediately after the disturbance caused by the butterfly (see Fig. 10B,C and Supplementary Video Clips VC8-VC11).

According to the Supplementary Results, in Hungary in the summer months of 2017 during the day the characteristic average wind speed was 2–5 km/h (Ref.^5^). Such wind speeds completely disrupt the upwelling air streams above sunlit zebra stripes.

## Discussion

According to the thermoregulation hypothesis, upwelling warm and downwelling cold air streams form above black and white stripes, which cool the body. Using schlieren imaging, we have shown that downwelling air streams do not form above our test surfaces imitating the curved back of zebras, and the upwelling warm streams are fed with colder air from the sides of the curved test surfaces. We have also demonstrated that an upward flowing warm air layer sticks to the curved surface, follows its curvature, secedes from the uppermost line of the cylindrical surface and rises vertically upwards. Consequently, the real airflow above sunlit zebras, as well as, black and white/grey horses corresponds to the schematic air streams in Fig. 1B–D, rather than that in Fig. 1A as hypothesized earlier.

The air streams above the test surface hsh1(8b8w)perp with 1 cm stripe width were surprisingly stable: their average lifespan *t* was one of the longest, while their covered distance *d*_covered_, mean speed *v*_mean_, maximum distance *d*_max_ and start–end distance *d*_se_ were the lowest or one of the lowest. This can be explained on the basis of our thermographic measurements: the dark brown stripes (similarly to the homogeneous dark brown surface hhbh) did not heat up as much as the black cattle hide. It is important to note that the warmer the surface, the more intense are the upwelling air streams, and thus, they generate a stronger entrainment which can weaken the stability of upwelling streams.

The air streams, we observed with schlieren imaging under calm (windless) laboratory conditions, cannot form in the field because of persistent winds^1^. Earlier field measurements^5^ showed that the higher the air temperature is, the higher are the wind speeds, and zero wind speed practically never occurs in the daytime (Supplementary Fig. S13). In our laboratory wind experiment we generated winds so weak that they were not measurable with our windmill. We found that even such very weak winds can easily blow off the upwelling air streams starting to develop (Fig. 10A), and thus, prevent the formation of any periodic or structured airflow pattern above any lamplit/sunlit surfaces. This makes the convection idea very difficult to sustain.

We have also observed that the edges of dark and bright stripes of the test surfaces can hinder the horizontal drift of upwelling air streams. However, this effect occurs only very close (1–2 cm) to the surface, and at 7 cm above it is negligible due to the strong turbulence. Long hair facilitates stream formation (Figs. 8C,D, 9C–G), because it helps the upwelling warm air stream to secede. Furthermore, long hair also hinders the horizontal drift of streams.

In our opinion, it is very questionable that the schlieren-observed air stream could cool the underlying warm surface better than a chaotic/turbulent airflow. Note, that the upwelling warm air streams observed by schlieren imaging obviously cool the warm surfaces, because they are replaced by cooler air from the sides. The darker the sunlit surface, the warmer is the air above it, which results in a faster rise of the upwelling warm streams. However, it has been demonstrated in field experiments that a darker fur heats an animal body much more than a brighter coat^2–5,7^. Air stream patterns can be periodic in the immediate vicinity of the striped surfaces under controlled laboratory conditions (Figs. 5E, 9), nevertheless, this stream pattern is not expected in any natural environment because it is blown off by even the weakest wind (Fig. 10).

Many studies^6–12^ have hypothesized that in direct sunlight a small-scale airflow may be set up between black and white stripes along the animal’s dorsum only with hot air rising above the warmer black and cooler descending above the colder white (Fig. 1A). In the opinion of Cobb and Cobb^7^, the irregularity of this flow may be increased, not only by the air rising from the black stripes and descending onto the white stripes, but also by the turbulence. The two flows moving in opposite directions abrade each other above the edges of every single stripe. However, our results clearly demonstrate that there are no descending air streams above sunlit white stripes, and the assumed convective eddies, composed of a series of adjacent upwelling and downwelling air streams, do not form above sunlit zebra stripes.

It is well known that zebra-striped coats do not attract tsetse flies^18,19^ and horseflies^1,20^ with respect to a homogeneous dark fur, and this is the most widely accepted hypothesis for zebra striping nowadays^21–23^. Cobb and Cobb^7^ suggested that the air next to the zebras’ skin may be sufficiently unstable to deter (biting) flies from landing. In our experiments, we observed very similar upwelling warm air streams and their behavior above both striped and homogeneous surfaces. Thus, if the air streams above the sunlit back of zebras could hinder the landing of flies, the same would be true also for that of any homogeneous (black, grey, brown, white) coloured ungulates. *Tabanus bromius*, a small horsefly from Europe, can definitely land on free-living zebras under sunny conditions^24^.

In this work we presented experimental evidence that a series of convective air eddies does not form above sunlit zebra stripes. Thus, the function of zebra stripes cannot be explained by the alleged cooling effect of such air eddies.

## Conclusions

Downwelling air streams do not form above the white stripes of sunlit zebra coats. The first reason for this is that narrow parallel upwelling warm streams above adjacent black stripes tend to join because of the known strong entrainment in turbulent streams. The second reason is that the heated-up air is streaming obliquely upward along the left and right side of the convex upper body part, and when both streams reach the uppermost horizontal back region, they fuse in a net vertical upward stream. The influence of stripes on the air stream formation (facilitating upwelling streams, hindering lateral stream drift) is negligible above a few centimeters from the surface. The borderlines of adjacent black and white stripes, furthermore longer hairs and plumicorns can more or less stabilize the start positions of upwelling warm air streams. In calm weather, upwelling air streams might form above sunlit zebra stripes, however, they are blown off by the weakest wind, or even by the slowest movement of the zebra. Since the upwelling warm air streams behave very similarly above homogeneous and striped sunlit coats under natural (windy) conditions, the streams above both coat types have practically the same cooling effect. The presented experimental results—together with the earlier field demonstrations by Caro^1^ and Horváth et al*.*^5^—disprove the widespread thermoregulation hypothesis involving air eddies as a sound explanation of the function of zebra stripes.

## Supplementary Information

Supplementary Information 1.

Supplementary Video 1.

Supplementary Video 2.

Supplementary Video 3.

Supplementary Video 4.

Supplementary Video 5.

Supplementary Video 6.

Supplementary Video 7.

Supplementary Video 8.

Supplementary Video 9.

Supplementary Video 10.

Supplementary Video 11.

## Data Availability

Our paper has the following Electronic Supplementary Material: Supplementary Results. Supplementary Figures S1-S13. Supplementary Video Clips VC1-VC11. Supplementary Tables S1-S12.
