# Supplementary material for: A new argument against cooling by convective air eddies formed above sunlit zebra stripes
Source: Sci Rep. 2021 Aug 4;11:15797. doi: 10.1038/s41598-021-95105-4 (PMC8339008; doi:10.1038/s41598-021-95105-4)
Supplement: Supplementary file 1 — Supplementary Information 1. [file 41598_2021_95105_MOESM1_ESM.docx]

**Supplementary Information**

for

**A new argument against cooling by convective air eddies formed above sunlit zebra stripes**

Ádám Pereszlényi, Dénes Száz, Imre M. Jánosi & Gábor Horváth^1,^*

1: Department of Biological Physics, ELTE Eötvös Loránd University, H-1117 Budapest,

Pázmány sétány 1, Hungary

*: correspondig author, e-mail: gh@arago.elte.hu

This file contains the following:

Supplementary Results

Schlieren images of air streams above smooth test surfaces

Schlieren images of air streams above hairy test surfaces

Typical summer wind speeds in Hungary

Supplementary Figures S1-S13

Supplementary Video Clips VC1-VC11

Supplementary Tables S1-S12

**Supplementary Results**

**Schlieren images of air streams above smooth test surfaces**

If the horizontal long axis of smooth monocoloured cylindrical test surfaces was perpendicular to the light beam, the formation of upwelling air streams was chaotic and the behaviour of these streams was unpredictable. As Fig. 5E shows, above the test surface ss1(8b7w) the stripe borders catalyze the formation of upwelling air streams which usually form at stripe edges. A newly emerged stream can slide along the uppermost horizontal line of the cylinder mantle, which drift is practically not affected by the stripes (Fig. 6, Supplementary Video Clip VC1). Above test surfaces ss3(3b2w), ss5(2b1w) and ss7.5(1b1w) with wider stripes (Figs. 5F-H) the catalyzing effect is weaker, because streams can also form within the stripes, not just at their borders. Similar effect was not observed above the surface ss0.5(15b15w) with 0.5 cm stripe width, probably because the stripes were thinner than a thermo/aerodynamically critical threshold.

The PCA analysis showed that *N*_min_ and Δ*I* positively correlated, while *d*_ave_ negatively correlated with these two variables (Supplementary Fig. S6). The variables from the upper window are independent of those from the lower window. The scatter (characterized by confidence ellipses) of variables of the homogeneous smooth white shw and grey shg test surfaces was larger than that of the striped surfaces in the lower window, while the scatter of the striped surfaces was larger than that of the homogeneous surfaces in the upper window. The largest scatter ellipse of the PCA plot belongs to the homogeneous smooth black surface shb and was between that of the homogeneous bright (shw and shg) and striped surfaces. Among the striped test surfaces, the scatter ellipse of surface ss1(8b7w) with 1 cm stripe width slightly separated from those the other ones, because of the variables of the lower window. The first two principal components explained the 70 % of the total variance (Supplementary Table S6).

The statistical analysis of air stream behaviour above the smooth test surfaces showed that surface ss1(8b7w) with 1 cm stripe width significantly differed from most of the other striped surfaces in almost all variables *t*, *d*_covered_, *v*_mean_, *d*_max_ and *d*_se_ (Supplementary Fig. S7, Supplementary Table S7). The lifespan *t* of streams above surface ss1(8b7w) was the highest and only surface ss3(3b2w) with the second largest *t* did not differ statistically (*p* = 0.394) from ss1(8b7w). While *t* of ss1(8b7w) was the longest, *v*_mean_, *d*_max_ and *d*_se_ of this surface were the lowest, and its *d*_covered_ was one of the lowest (Supplementary Fig. S7). Furthermore, ss1(8b7w) had the most outliers, the lifespan *t* of which was much longer than that of other test surfaces. This means that the most stable upwelling air streams formed above ss1(8b7w). These findings support our previous observation that the stripe borders catalyze the stream formation and this effect is the strongest above surface ss1(8b7w). These suggest that the stripe borders can hinder the horizontal drift of upwelling air streams. For some air current this hindering is true (the tendentious path of the upwelling stream is vertical), however in other instances this effect cannot be seen.

According to the statistical analysis, the homogeneous white shw and grey shg test surfaces did not differ significantly from the other test surfaces (Supplementary Table S7), which finding seems to contradict the boxplots of variables (Supplementary Fig. S7). For example, considering variable *d*_max_, the difference between ss3(3b2w) and ss5(2b1w) is significant with *p* = 0.00814, while the differences between ss3(3b2w) and shw, or ss3(3b2w) and shg are not significant with *p* = 1 and *p* = 0.103, respectively (Supplementary Fig. S7B, Supplementary Table S7). This paradox is resolved by the fact that the number of air streams is much smaller above the brighter surfaces shw and shg than above the darker and striped ones (Supplementary Fig. S7F). Note that the air stream behaviour was analyzed only in the lower window of the schlieren images/sequences and in this analysis only streams with lifespan *t* > 1 second were involved (Supplementary Fig. S7F). In the upper window such analysis was not performed due to the too short lifespan *t* of streams.

According to the PCA analysis of air stream behaviour above the smooth test surfaces, the mean speed *v*_mean_, start-end distance *d*_se_, maximum distance *d*_max_ and covered distance *d*_covered_ correlated positively, while the lifespan *t* was independent of these variables (Supplementary Fig. S8). Test surface ss1(8b7w) with 1 cm stripe width slightly separated from the others, because *t* of streams above ss1(8b7w) was longer, while *d*_se_, *d*_max_, *d*_covered_ and *v*_mean_ were smaller than those above other surfaces. Supplementary Table S8 contains the total variance explained by each component.

**Schlieren images of air streams above hairy test surfaces**

Above the hairy test surface hsh1(8b8w)perp (Fig. 9A) the stream formation and behaviour were similar to those above the smooth test surface ss1(8b7w) with 1 cm stripe width, but the contrast of streams in schlieren images and intensity of stream formation above the former were lower than above the latter. The reason for this was that the dark brown horse hide used for the dark stripes (used also for the homogeneous dark brown test surface hhbh) did not warm up as much as the smooth black surface. The stream formation and behaviour above surfaces hhwc (with short hair, Fig. 8A) and hhbh (Fig. 8B) were similar to those above the bright smooth homogeneous surfaces shg and shw. However, the average black stripe width of the zebra hide hsz(8b7w)perp (Fig. 9B) was close to 1 cm, the stream formation and behaviour were more similar to the homogeneous short-haired surfaces hhbh and hhwc, because the shape of the stripes were not straight and this might have distracted the stream formation, furthermore the stripe borders were not as sharp as those of the smooth striped surfaces.

According to the statistical analysis of hairy test surfaces, in the lower window *N*_min_(lower) was the smallest above surfaces hsz(8b7w)perp, hhwc and hhbh (Supplementary Fig. S9) differing significantly from the other hairy surfaces (Supplementary Table S9). *N*_min_(lower) above surface hsh1(8b8w)perp with 1 cm stripe width was between those of the short- and long-haired surfaces. In the lower window, the most upwelling air streams formed above the homogeneous black surface hhbc which differed highly significantly from all other hides, except for surface hsc1(7b7w)perp with 1 cm stripe width (*p* = 0.4335). These results also suggest that the stripe borders facilitate the formation of upwelling warm air streams, and the long hairs catalyze this formation even more.

In the upper window, *N*_min_(upper) of streams above surface hsh1(8b8w)perp with 1 cm stripe width did not differ significantly from that of surfaces hsz(8b7w)perp, hhwc and hhbh, but differed highly significantly from those above all other surfaces (Supplementary Table S9). This means that the upwelling air streams were more stable above the long-haired surfaces than above the short-haired ones and remained 7 cm above the surface. Furthermore, the short-haired surfaces hsh1(8b8w)perp, hsz(8b7w)perp, hhwc and hhbh did not warm up as much as the long-haired black cattle hide (Fig. 4, Supplementary Table S4). Thus, the influence of stripes was weaker at 7 cm above the short-haired surfaces. It is noticeable that in the upper window the most upwelling air streams with maximum *N*_min_(upper) formed above the surface hsc1(8b7w)par, when the horizontal stripes were parallel to the light beam (Supplementary Fig. S9). On the other hand, *d*_ave_(upper) of hsc1(8b7w)par was the smallest compared to the other hairy surfaces. This suggests a negative correlation between *N*_min_ and *d*_ave_. Δ*I* correlates positively with *N*_min_. Considering variables *N*_min_(upper), *d*_ave_(upper) and Δ*I*(upper), the grey cattle hides hsgc3(3s2L)perp and hhgc were more similar to the bright surfaces hhwc, hhbh and hsz(8b7w)perp (Supplementary Fig. S9).

According to the PCA analysis of the hairy test surfaces *N*_min_ positively correlated with Δ*I*, while both variables negatively correlated with *d*_ave_ (Supplementary Fig. S10) as for smooth surfaces. In the lower window of the schlieren images/sequences above hairy surfaces the correlation between *N*_min_(lower) and Δ*I*(lower) was not as strong as in the case of smooth surfaces (Supplementary Figs. S6, S10). Considering *N*_min_ and *d*_ave_ in both upper and lower windows, furthermore Δ*I* in the lower window, the short-haired test surfaces hsz(8b7w)perp, hhwc and hhbh separated from the others, while the horse hide hsh1(8b8w)perp with 1 cm stripe width was between them (Supplementary Fig. S9). The first two principal components explained the 78.1 % of the total variance (Supplementary Table S10).

On the basis of the statistical analysis of air stream behaviour, the lifespans *t* of streams above test surfaces hsh1(8b8w)perp, hsc1(7b7w)perp and hsc3(3b2w)perp were the longest, while their mean speeds *v*_mean_ were the lowest (Supplementary Fig. S11). These three surfaces did not differ significantly from each other in any variables (Supplementary Table S11). Although *d*_covered_, *v*_mean_, *d*_max_ and *d*_se_ of air streams above the brown horse hide hhbh were the highest (Supplementary Fig. S11) – which was expected on the basis of the previous observations: this surface is smooth, homogeneous and warmer than the white cattle hide hhwc –, hhbh did not differ statistically from hhwc in any variables (Supplementary Table S11). Supplementary Figure S11F shows the number of upwelling air streams with lifespan *t* > 1 second used in the statistical analysis. As expected, the number of streams was the lowest above surfaces hhwc and hhbh (Supplementary Fig. S11).

The PCA analysis of air stream behaviour above hairy surfaces showed that the relation of variables was similar to that above smooth surfaces. However, *v*_mean_, *d*_se_ and *d*_max_ less correlated, and *d*_covered_ correlated more with the lifespan *t* than with the other variables, independently of *v*_mean_ and *d*_se_ (Supplementary Fig. S12). Supplementary Table S12 shows the total variance explained by each component. The centre of the fitted ellipses are at the same area of the PCA plot, which suggests that the properties of the many short-living streams were similar, independently of the type of hairy surfaces, while the shape of the ellipses are determined by the relatively few, but long-living and/or mobile streams (this is also true for the smooth test surfaces, Supplementary Fig. S8). *t* of streams above surfaces hhwc and hhbh was short, but *v*_mean_, *d*_se_ and *d*_max_ were diverse. Above the test surfaces hsh1(8b8w)perp and hsc3(3b2w)perp with overlapping ellipses, *d*_covered_ and *t* were larger and more diverse than above other surfaces. Considering any variable, there was no statistical difference between the two orientations (long axis perpendicular or parallel to the light beam) of surface hsc1(8b7w)par (Supplementary Table S11), and in the PCA plot the ellipses belonging to both orientations completely overlapped (Supplementary Fig. S12). However, when the cylinder’s horizontal long axis was parallel to the light beam, most of the streams moved to the cylinder’s uppermost (middle) line, while when the cylinder’s long axis was perpendicular to the light beam, such tendency towards the middle did not occur.

**Typical summer wind speeds in Hungary**

Supplementary Figure S13A shows the daily change of the wind speed *w* measured by Horváth *et al*. (2018) between 10 June and 19 September 2017 in a summer field experiment. We can clearly see the typical meteorological phenomenon that after sunrise the average wind speed increased and culminated (reached its maximum) at around 15:00-16:00 hours. The range of the average wind speed was 2 km/h ≤ *w*_average_ ≤ 5 km/h between sunrise and sunset. Supplementary Figure S13B displays the daily change of the air temperature *T*_air_ measured during the same field experiment. The daily variation of *T*_air_ had the same pattern as *w*. When *T*_air_ was the highest, *w*_average_ was between 3 and 6 km/h. According to Supplementary Fig. S13C, the higher the air temperature, the larger the wind speed. This positive correlation between *w* and *T*_air_ is highly significant on the basis of the Spearman rho correlation test (p < 0.0001, S = 1.6832·10^11^, rho = 0.4733744).

**Supplementary Figures with Captions**


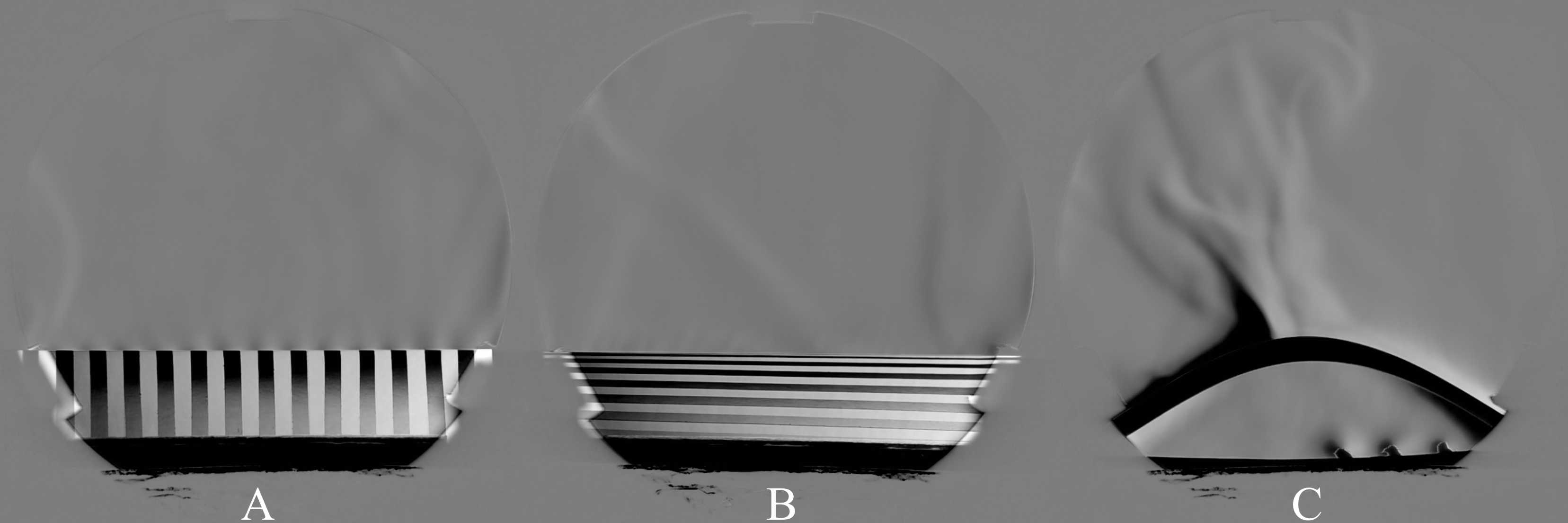


**Supplementary Figure S1.** Schlieren photographs of airflows above test surfaces in three different setups. The horizontal long axis of the cylindrical test surface was perpendicular (A, B) or parallel (C) to the collimated horizontal light beam illuminating the target area, while the stripes were perpendicular (A) or parallel (B) to the cylinder’s long axis.


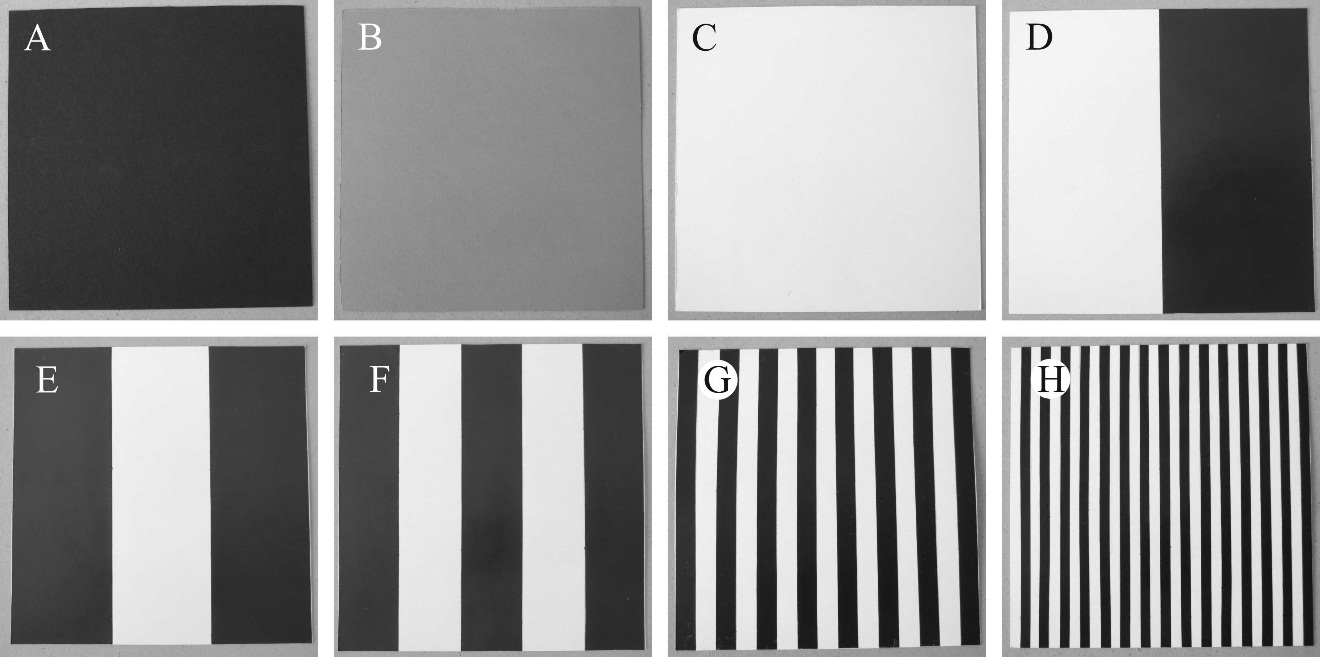


**Supplementary Figure S2.** The 8 smooth test surfaces (15 cm × 15 cm) used in the schlieren and thermography measurements. (A-C) Homogeneous. (A) shb: black. (B) shg: grey. (C) shw: white. (D-H) Striped. (D) ss7.5(1b1w): 1 black + 1 white stripe with 7.5 cm stripe width. (E) ss5(2b1w): 2 black + 1 white stripes with 5 cm stripe width. (F) ss3(3b2w): 3 black + 2 white stripes with 3 cm stripe width. (G) ss1(8b7w): 8 black + 7 white stripes with 1 cm stripe width. (H) ss0.5(15b15w): 15 black + 15 white stripes with 0.5 cm stripe width.


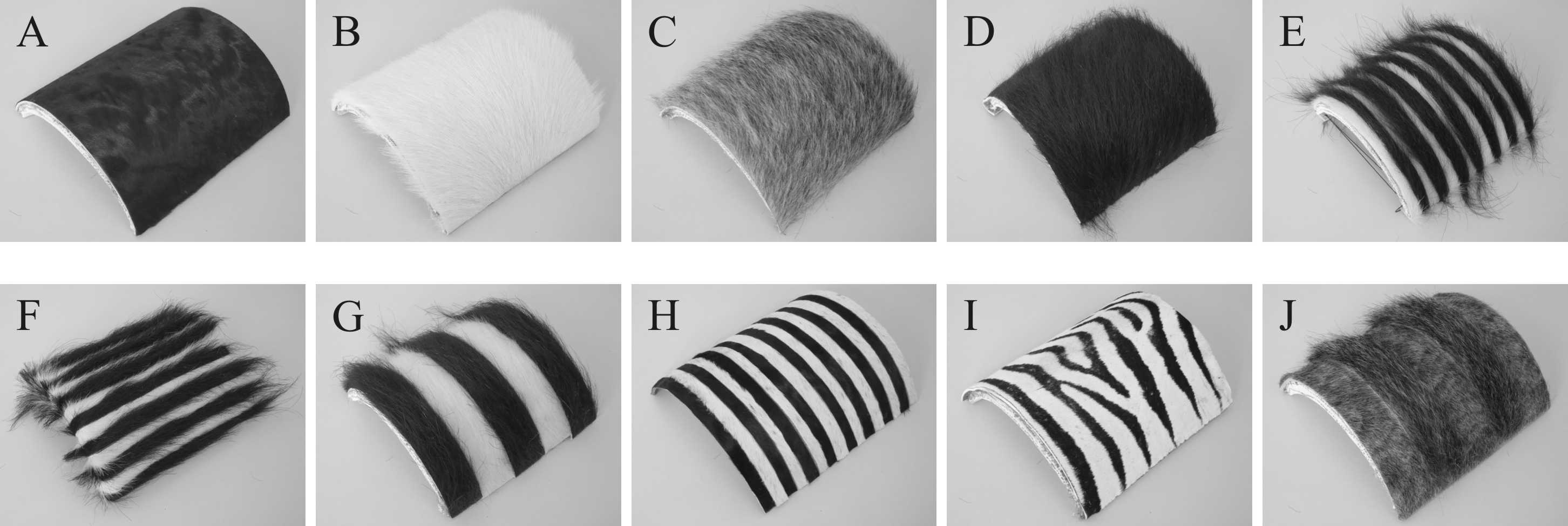


**Supplementary Figure S3.** The 10 horizontal cylindrical hairy test surfaces (length: 15 cm, radius: 7 cm) used in the schlieren and thermography measurements. (A-D) Homogeneous. (A) hhbh: dark brown horse hide. (B) hhwc: white cattle hide. (C) hhgc: grey cattle hide. (D) hhbc: black cattle hide. (E-J) Striped. (E) hsc1(7b7w)perp: cattle hide with 7 long-haired black stripes + 7 short-haired white stripes perpendicular to the cylinder’s horizontal long axis. (F) hsc1(8b7w)par: cattle hide with 8 long-haired black stripes + 7 short-haired white stripes parallel to the cylinder’s long axis. (G) hsc3(3b2w)perp: cattle hide with 3 long-haired black stripes + 2 short-haired white stripes perpendicular to the cylinder’s long axis. (H) hsh1(8b8w)perp: horse hide with 8 short-haired black stripes + 8 short-haired white stripes perpendicular to the cylinder’s long axis. (I) hsz(8b7w)perp: zebra hide with 8 short-haired black stripes + 7 short-haired white stripes perpendicular to the cylinder’s long axis. (J) hsgc3(3s2L)perp: grey cattle hide with 3 short-haired stripes + 2 long-haired stripes.


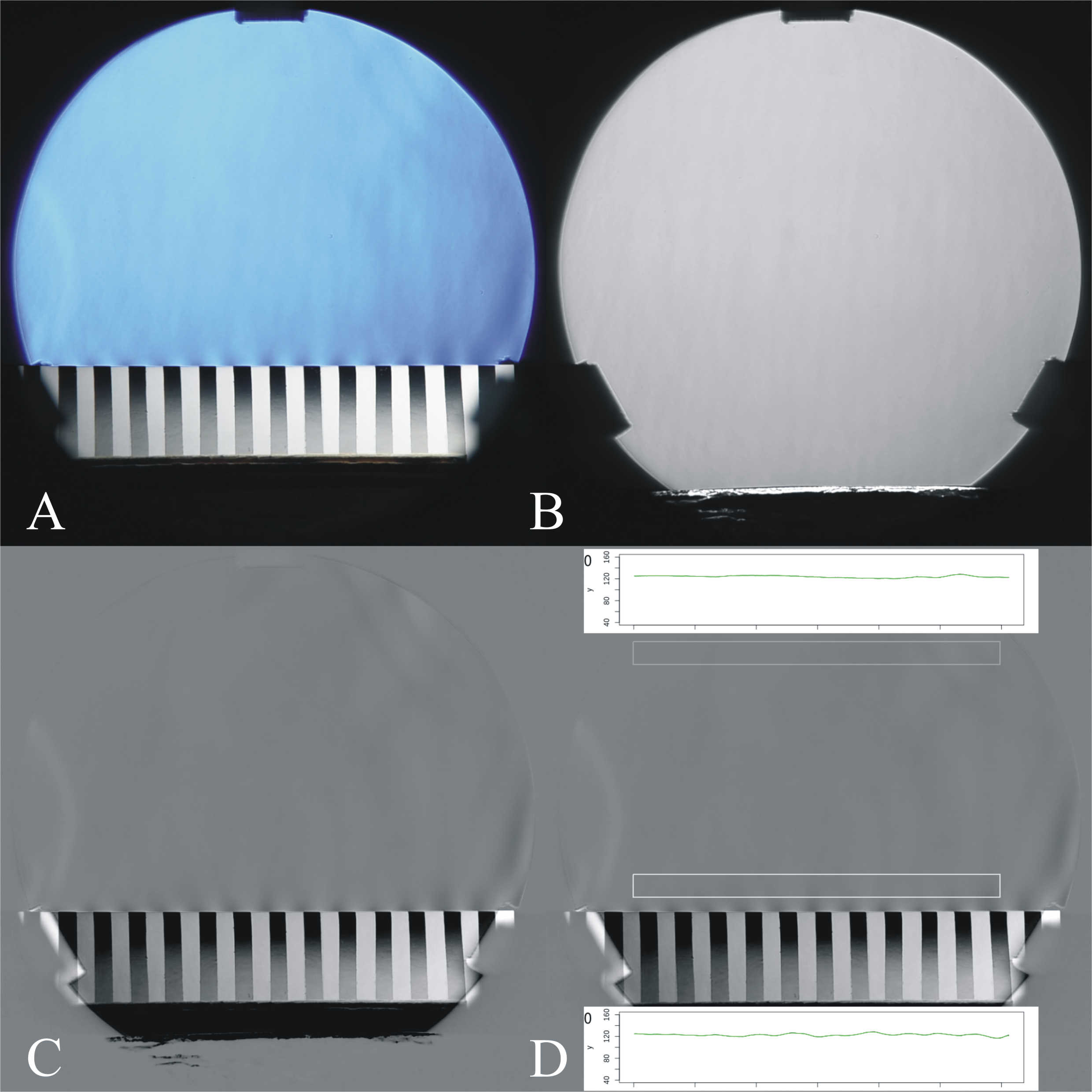


**Supplementary Figure S4.** (A) Original (coloured) schlieren image of the air layer above a lamplit cylindrical striped test surface when the curved stripes are parallel and the cylinder’s horizontal long axis is perpendicular to the light beam. (B) Averaged schlieren image of the background without the test surface. (C) Filtered schlieren image (the averaged background image is subtracted from the original image converted to a grey-shaded one). (D) Upper and lower horizontally elongated rectangular windows (with a bright perimeter) in the filtered schlieren image of a test surface together with the plot of the averaged pixel intensity *I*(*x*) along the horizontal axis *x* of the rectangles. The number *N*_min_ of local minima of *I*(*x*) depicted by vertical lines is given in the top left corner of the plots.


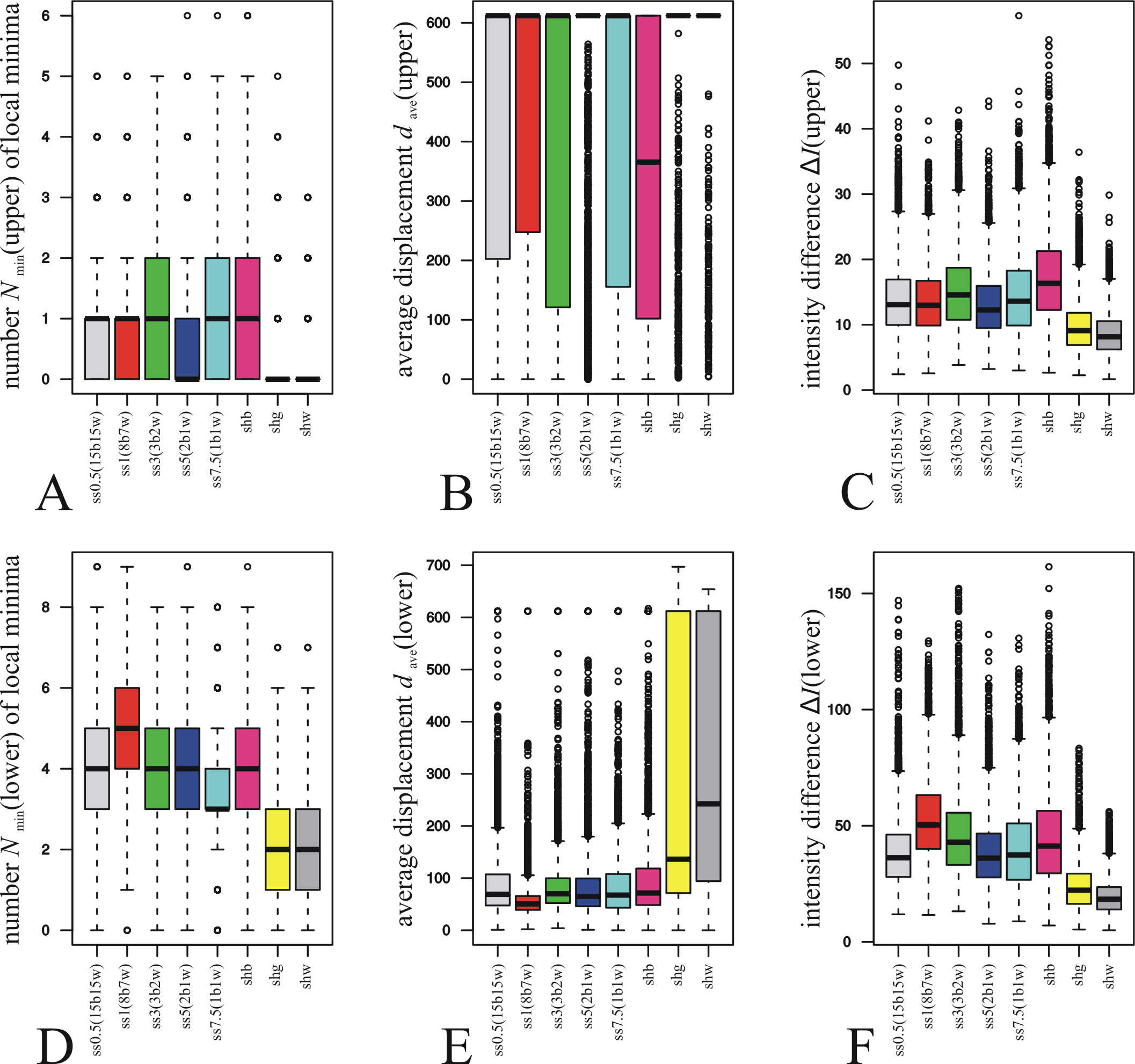


**Supplementary Figure S5.** Minimum, first quartile, median, third quartile, maximum and outliers (extrema: circles) of variables number *N*_min_(upper) of local minima of *I*(*x*) (A), average displacement *d*_ave_(upper) of each local minimum (B), intensity difference Δ*I*(upper) (C), *N*_min_(lower) (D), *d*_ave_(lower) (E) and Δ*I*(lower) (F) measured in the upper and lower windows for the smooth test surfaces. The long axis of both homogeneous and striped cylindrical test surfaces was perpendicular and the curved stripes were parallel to the light beam. Supplementary Table S5 contains the results of the Wilcoxon rank sum test applied for this dataset.


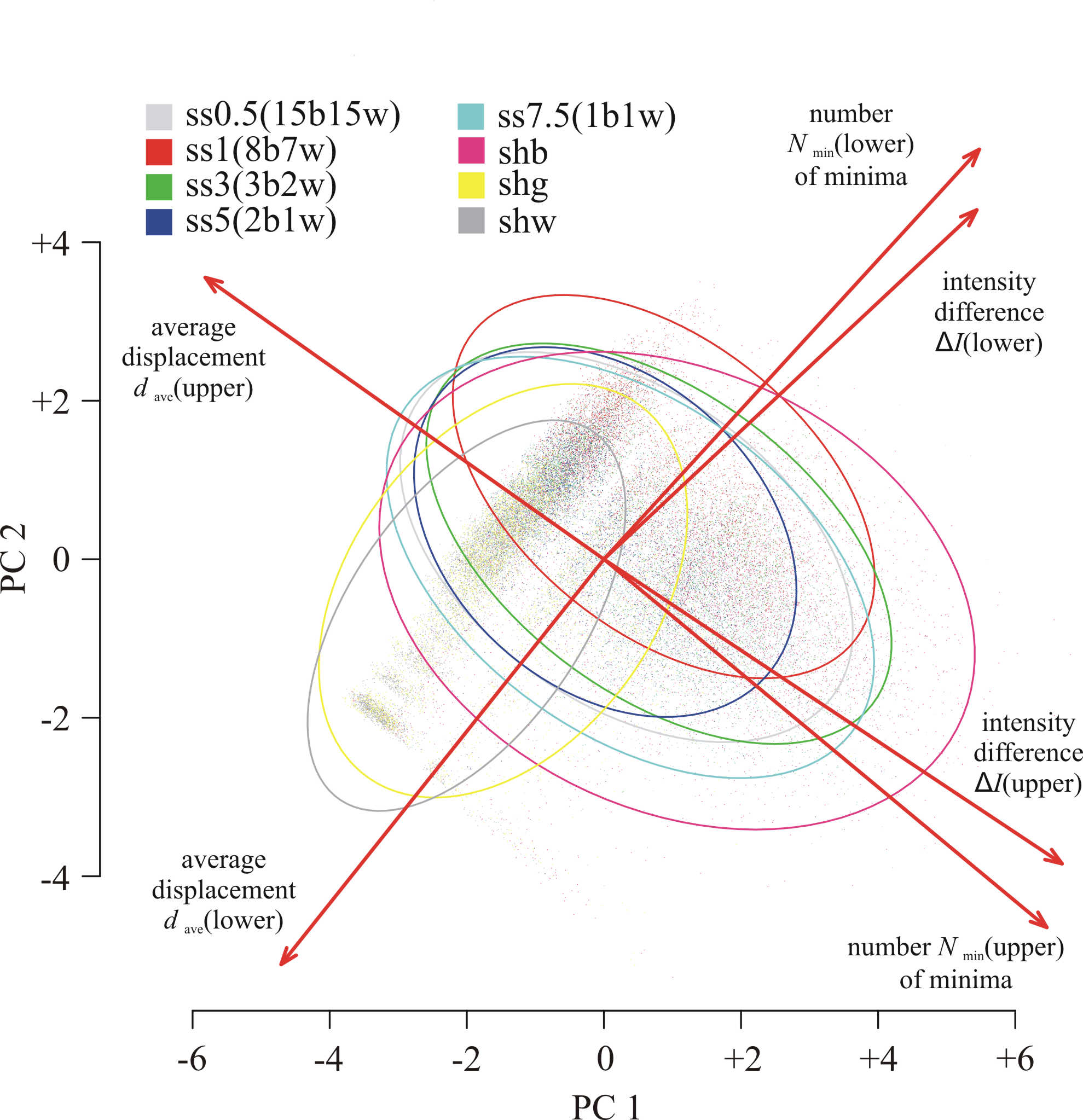


**Supplementary Figure S6.** PCA plot of the first two principal components of variables *N*_min_(upper), *d*_ave_(upper), Δ*I*(upper), *N*_min_(lower), *d*_ave_(lower) and Δ*I*(lower) measured in the upper and lower windows, using the data of the smooth test surfaces. Ellipses are fitted to the scores with 95 % confidence interval. The long axis of both homogeneous and striped cylindrical test surfaces was perpendicular and the curved stripes were parallel to the light beam. Supplementary Table S6 contains the total variance explained by each component.


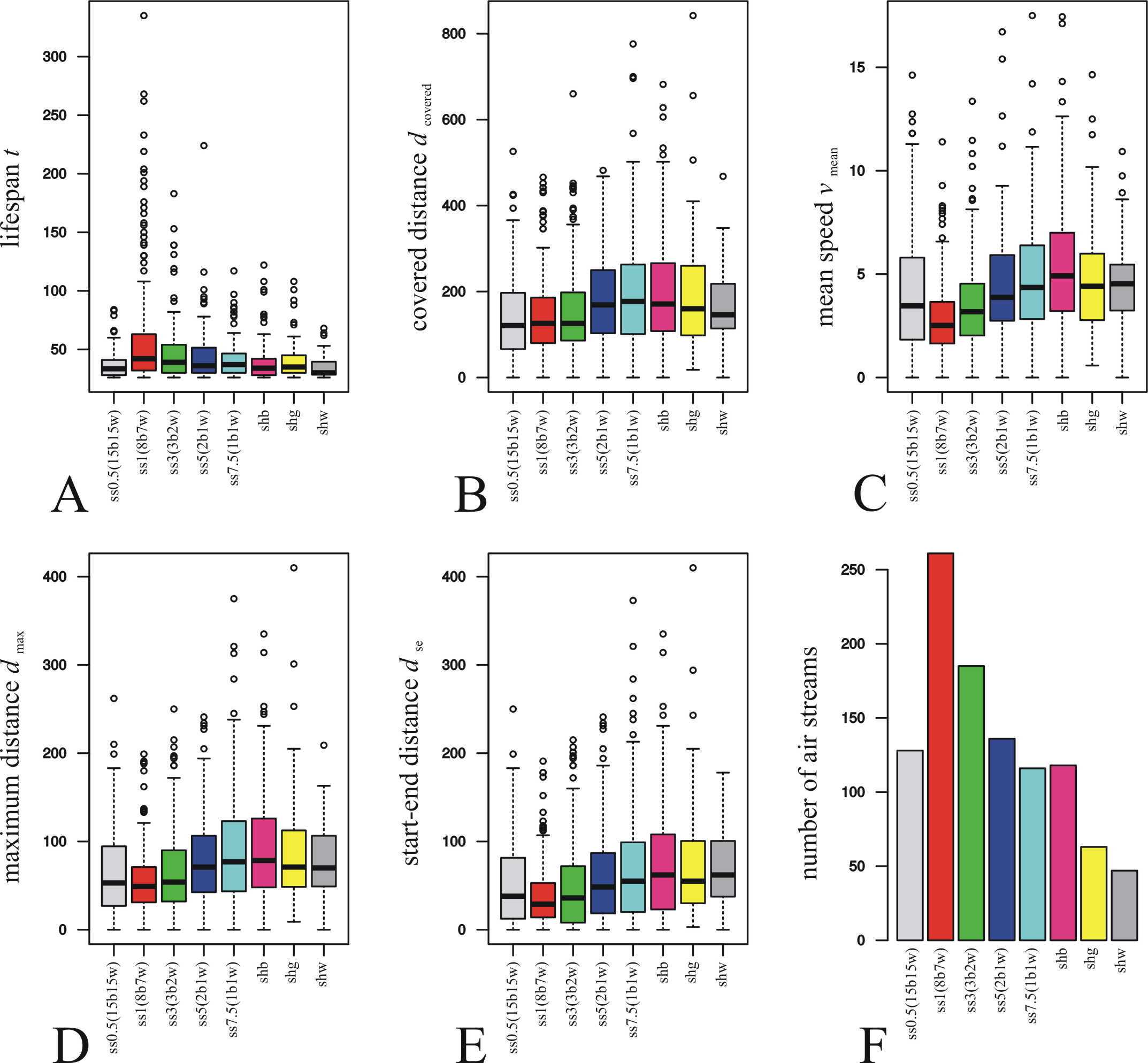


**Supplementary Figure S7.** Minimum, first quartile, median, third quartile, maximum and outliers (extrema: circles) of variables lifespan *t* (A), covered distance *d*_covered_ (B), mean speed *v*_mean_ (C), maximum distance *d*_max_ (D) and start-end distance *d*_se_ (E) measured in the lower windows for the smooth test surfaces. Only air streams with lifespan *t* > 1 second were considered; F shows the number of these streams for the different test surfaces. The long axis of the cylindrical test surfaces was perpendicular and the curved stripes were parallel to the light beam. Supplementary Table S7 contains the results of the Wilcoxon rank sum test applied for this dataset.


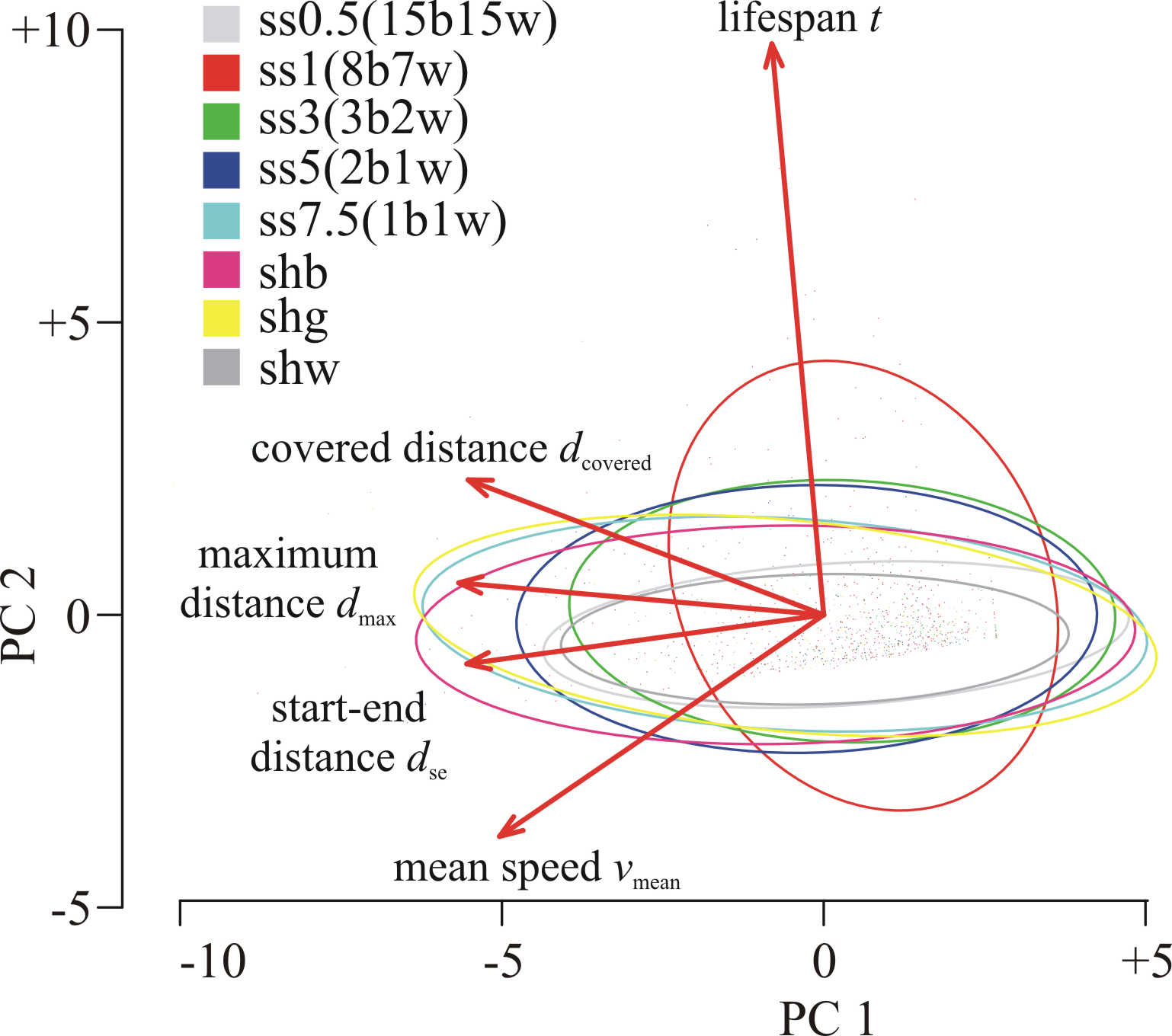


**Supplementary Figure S8.** PCA plot of the first two principal components of variables lifespan *t*, covered distance *d*_covered_, mean speed *v*_mean_, maximum distance *d*_max_ and start-end distance *d*_se_, measured in the lower windows, using the data of the smooth test surfaces. Only air streams with lifespan *t* > 1 second were considered. Ellipses are fitted to the scores with 95 % confidence interval. The long axis of both homogeneous and striped cylindrical test surfaces was perpendicular and the curved stripes were parallel to the light beam. Supplementary Table S8 contains the total variance explained by each component.


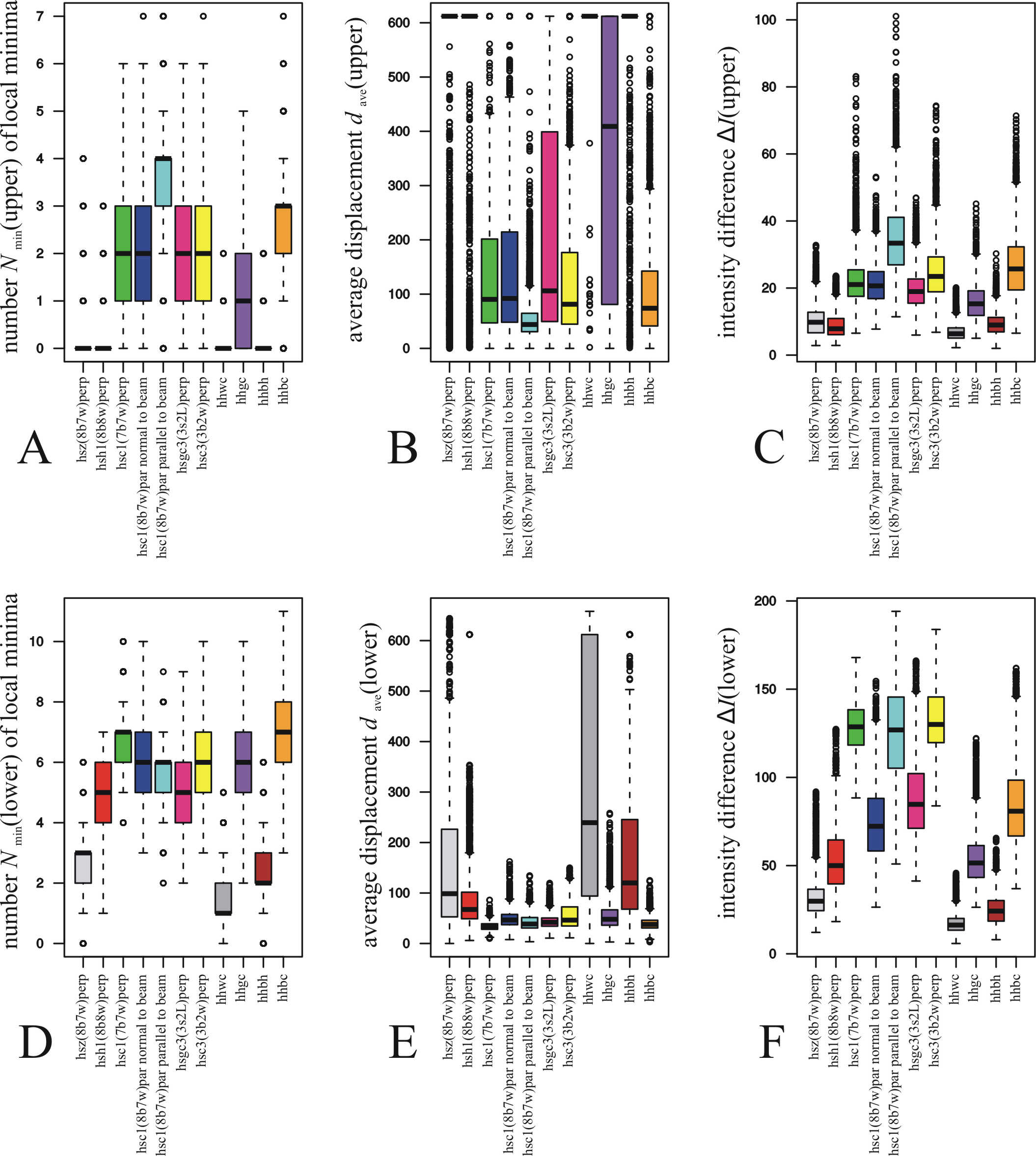


**Supplementary Figure S9.** Minimum, first quartile, median, third quartile, maximum and outliers (extrema: circles) of variables *N*_min_ (A), *d*_ave_(upper) (B), Δ*I*(upper) (C), *N*_min_(lower) (D), *d*_ave_(lower) (E) and Δ*I*(lower) (F) measured in the upper and lower windows for the hairy test surfaces. The long axis of the cylindrical striped test surfaces was perpendicular and the curved stripes were parallel to the light beam for hsz(8b7w)perp, hsh1(8b8w)perp, hsc1(7b7w)perp, hsgc3(3s2L)perp, hsc3(3b2w)perp. For hsc1(8b7w)par the horizontal stripes were parallel and perpendicular to the light beam. For hhwc, hhgc, hhbh and hhbc the long axis of the cylindrical homogeneous test surfaces was perpendicular to the light beam. Supplementary Table S9 contains the results of the Wilcoxon rank sum test applied for this dataset.


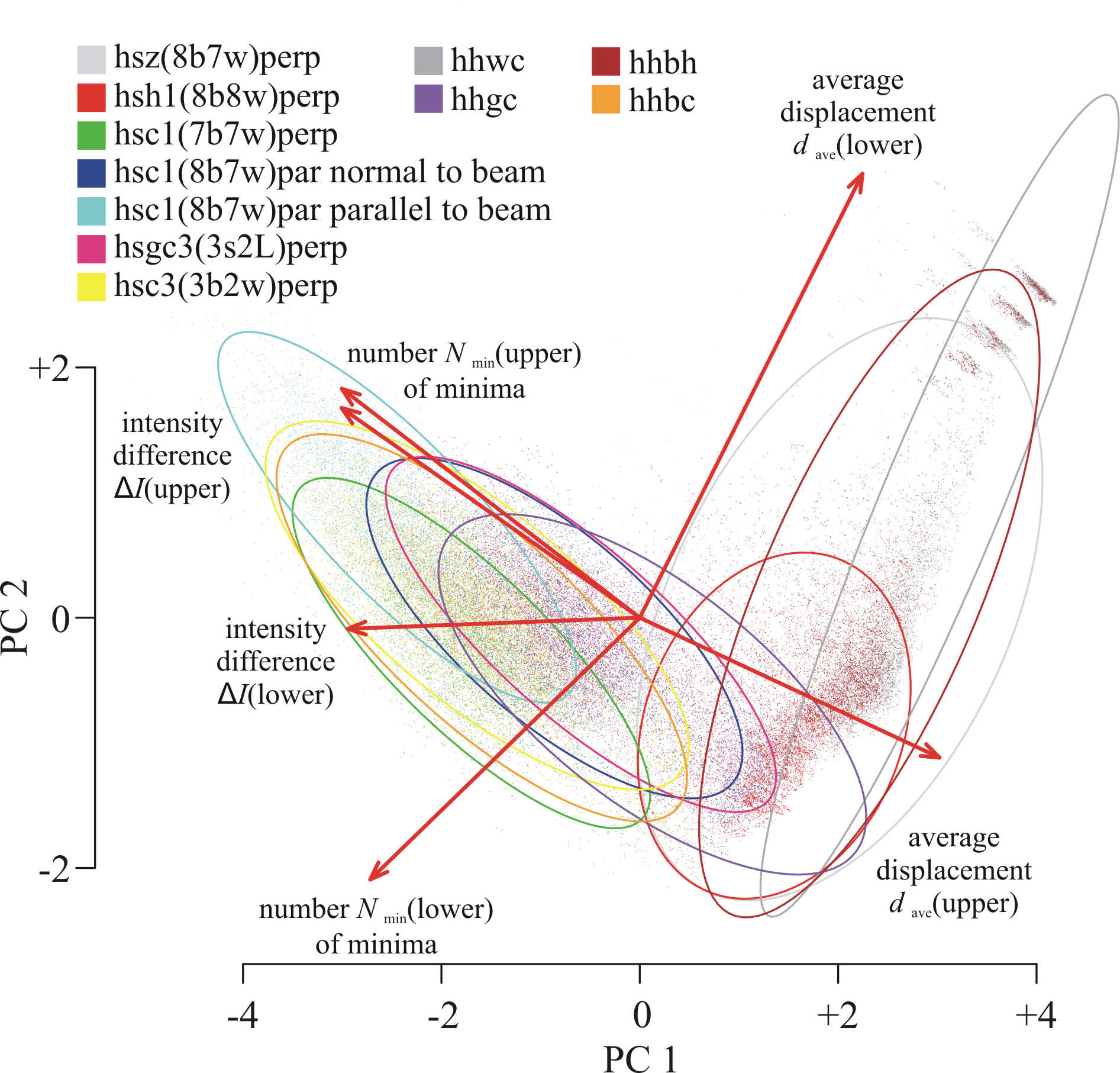


**Supplementary Figure S10.** PCA plot of the first two principal components of variables *N*_min_(upper), *d*_ave_(upper), Δ*I*(upper), *N*_min_(lower), *d*_ave_(lower) and Δ*I*(lower) measured in the upper and lower windows, using the data of the hairy test surfaces. Ellipses are fitted to the scores with 95 % confidence interval. The long axis of the cylindrical striped test surfaces was perpendicular and the curved stripes were parallel to the light beam for hsz(8b7w)perp, hsh1(8b8w)perp, hsc1(7b7w)perp, hsgc3(3s2L)perp, hsc3(3b2w)perp. For hsc1(8b7w)par the horizontal stripes were parallel and perpendicular to the light beam. For hhwc, hhgc, hhbh and hhbc the long axis of the cylindrical homogeneous test surfaces was perpendicular to the light beam. Supplementary Table S10 contains the total variance explained by each component.


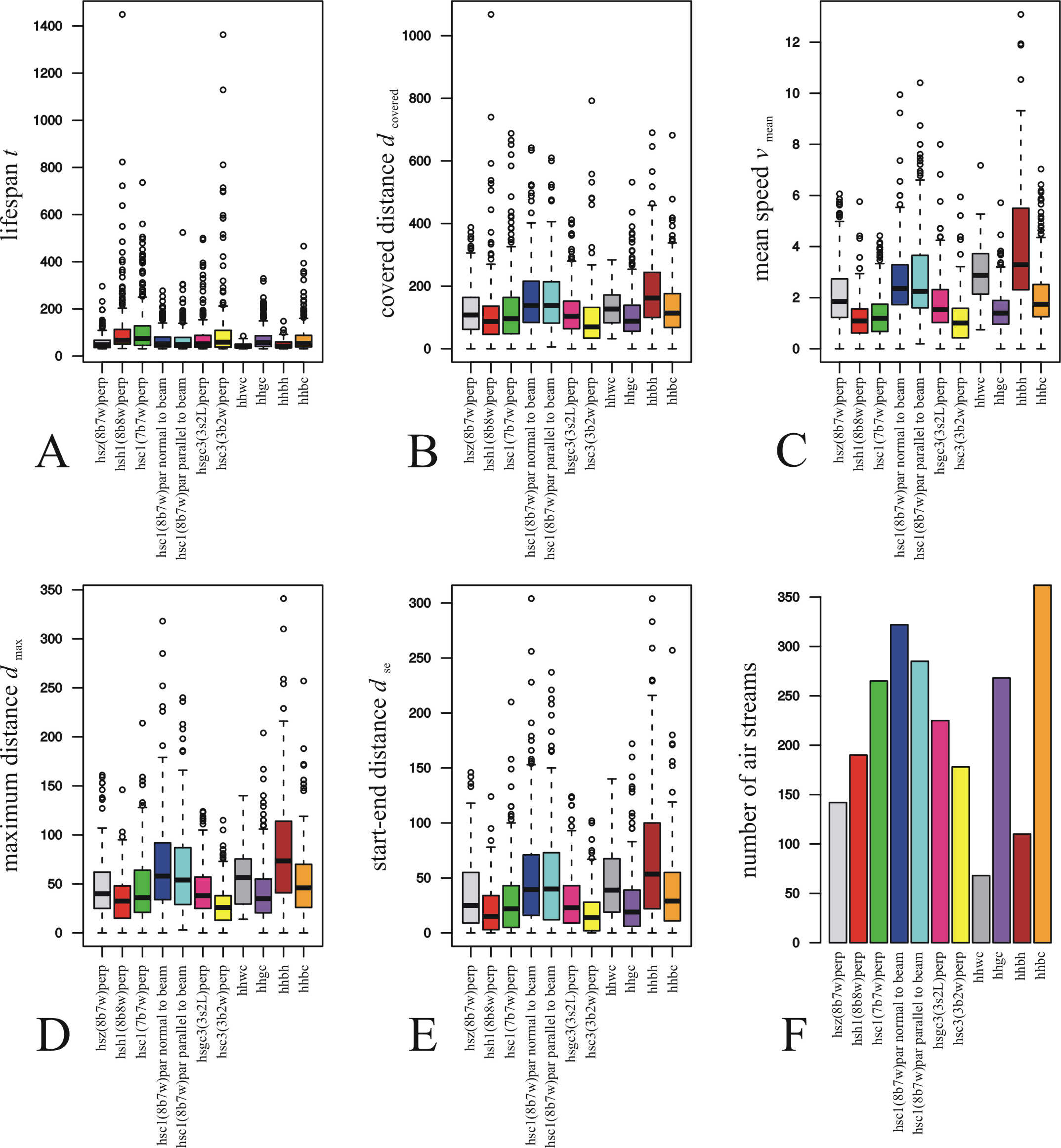


**Supplementary Figure S11.** Minimum, first quartile, median, third quartile, maximum and outliers (extrema: circles) of variables *t* (A), *d*_covered_ (B), *v*_mean_ (C), *d*_max_ (D) and *d*_se_ (E) measured in the lower windows for the hairy test surfaces. Only air streams with lifespan *t* > 1 second were considered; F shows the number of these streams for the different test surfaces. The long axis of the cylindrical striped test surfaces was perpendicular and the curved stripes were parallel to the light beam for hsz(8b7w)perp, hsh1(8b8w)perp, hsc1(7b7w)perp, hsgc3(3s2L)perp, hsc3(3b2w)perp. For hsc1(8b7w)par the horizontal stripes were parallel and perpendicular to the light beam. For hhwc, hhgc, hhbh and hhbc the long axis of the cylindrical homogeneous test surfaces was perpendicular to the light beam. Supplementary Table S11 contains the results of the Wilcoxon rank sum test applied for this dataset.


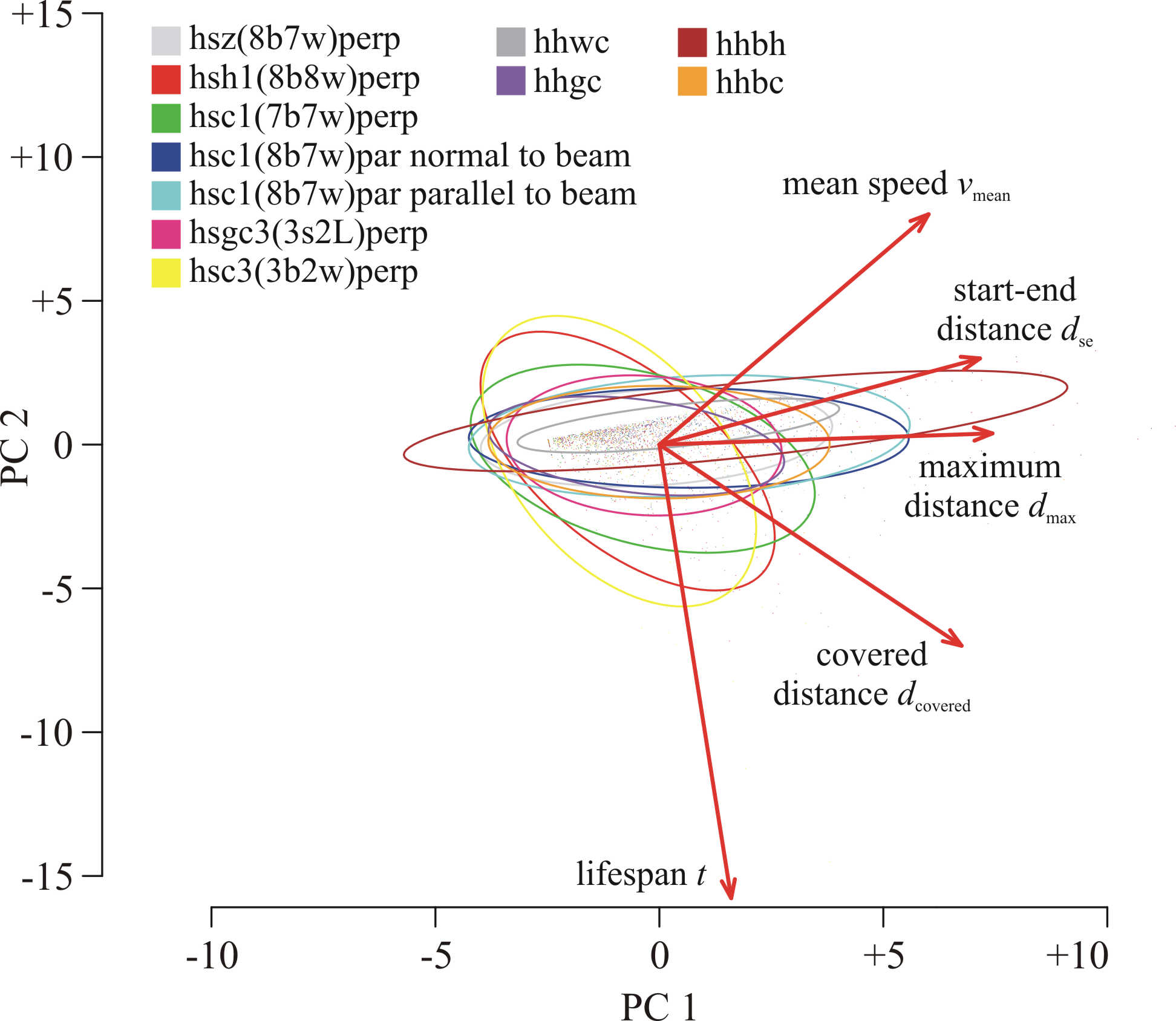


**Supplementary Figure S12.** PCA plot of the first two principal components of variables *t*, *d*_covered_, *v*_mean_, *d*_max_ and *d*_se_ measured in the lower windows, using the data of the hairy test surfaces. Only air streams with lifespan *t* > 1 second were considered. Ellipses are fitted to the scores with 95 % confidence interval. The long axis of the cylindrical striped test surfaces was perpendicular and the curved stripes were parallel to the light beam for hsz(8b7w)perp, hsh1(8b8w)perp, hsc1(7b7w)perp, hsgc3(3s2L)perp, hsc3(3b2w)perp. For hsc1(8b7w)par the horizontal stripes were parallel and perpendicular to the light beam. For hhwc, hhgc, hhbh and hhbc the long axis of the cylindrical homogeneous test surfaces was perpendicular to the light beam. Supplementary Table S12 contains the total variance explained by each component.


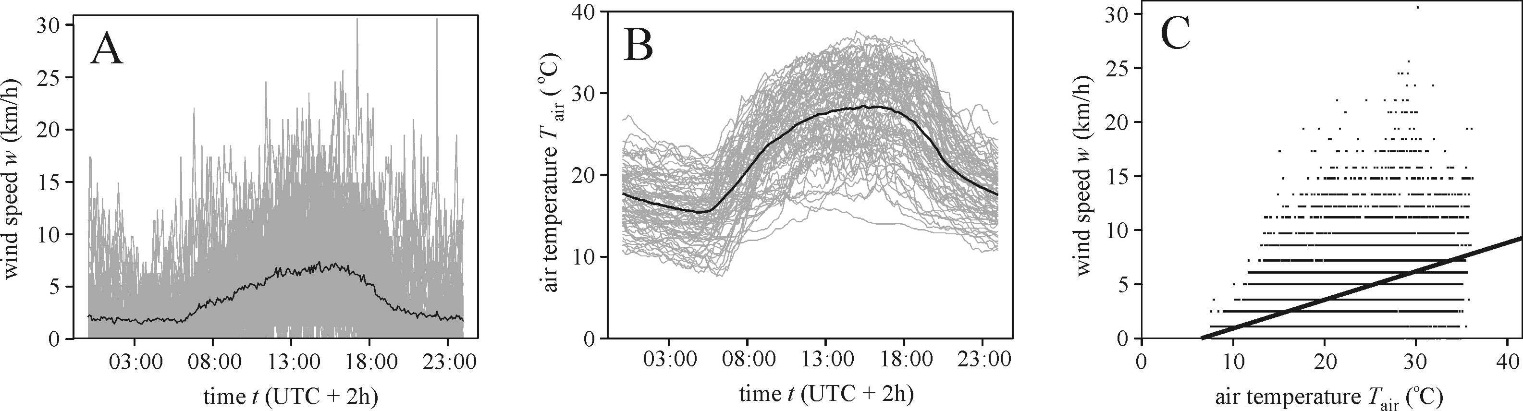


**Supplementary Figure S13.** (A) Daily change of the wind speed *w* (km/h) (grey) and its average (black) versus time *t* (= UTC + 2 h) measured by Horváth *et al*. (2018) between 10 June and 19 September 2017 in a field experiment. (B) Daily change of the air temperature *T*_air_ (^o^C) (grey) and its average (black) measured during the same field experiment. (C) Wind speed *w* (km/h) as a function of the air temperature *T*_air_ (^o^C) in the field experiment. The tilted straight line is the linear fit to the data (dots).

**Supplementary Video Clips**

**Supplementary Video Clip VC1**: Schlieren video sequence of test surface ss1(8b7w). The curved stripes of the cylindrical test surface were parallel and the cylinder’s horizontal long axis was perpendicular to the light beam. The plots show the averaged pixel intensity *I*(*x*) along the horizontal axis *x* of the upper and lower horizontally elongated rectangular windows. The number *N*_min_ of local minima of *I*(*x*) depicted by vertical lines is given in the top left corner of the plots.

**Supplementary Video Clip VC2**: Schlieren video sequence of test surface ss1(8b7w). The curved stripes of the cylindrical test surface were perpendicular and the cylinder’s horizontal long axis was parallel to the light beam. The plots show the averaged pixel intensity *I*(*x*) along the horizontal axis *x* of the upper and lower horizontally elongated rectangular windows. The number *N*_min_ of local minima of *I*(*x*) depicted by vertical lines is given in the top left corner of the plots.

**Supplementary Video Clip VC3**: Schlieren video sequence of test surface ss1(8b7w). The horizontal stripes and the cylinder’s horizontal long axis were parallel to the light beam. The plots show the averaged pixel intensity *I*(*x*) along the horizontal axis *x* of the upper and lower horizontally elongated rectangular windows. The number *N*_min_ of local minima of *I*(*x*) depicted by vertical lines is given in the top left corner of the plots.

**Supplementary Video Clip VC4**: Schlieren video sequence of test surface ss1(8b7w) in the wind experiment. The curved stripes of the cylindrical test surface were parallel and the cylinder’s horizontal long axis was perpendicular to the light beam.

**Supplementary Video Clip VC5**: Schlieren video sequence of test surface hsz(8b7w)perp in the wind experiment. The curved stripes of the cylindrical test surface were parallel and the cylinder’s horizontal long axis was perpendicular to the light beam.

**Supplementary Video Clip VC6**: Schlieren video sequence of test surface hsh1(8b8w)perp in the wind experiment. The curved stripes of the cylindrical test surface were parallel and the cylinder’s horizontal long axis was perpendicular to the light beam.

**Supplementary Video Clip VC7**: Schlieren video sequence of test surface hhbc in the wind experiment. The cylinder’s horizontal long axis was perpendicular to the light beam.

**Supplementary Video Clip VC8**: Schlieren video sequence of test surface ss1(8b7w) in the butterfly experiment. The curved stripes of the cylindrical test surface were parallel and the cylinder’s horizontal long axis was perpendicular to the light beam.

**Supplementary Video Clip VC9**: Schlieren video sequence of test surface hsz(8b7w)perp in the butterfly experiment. The curved stripes of the cylindrical test surface were parallel and the cylinder’s horizontal long axis was perpendicular to the light beam.

**Supplementary Video Clip VC10**: Schlieren video sequence of test surface hsh1(8b8w)perp in the butterfly experiment. The curved stripes of the cylindrical test surface were parallel and the cylinder’s horizontal long axis was perpendicular to the light beam.

**Supplementary Video Clip VC11**: Schlieren video sequence of test surface hhbc in the butterfly experiment. The cylinder’s horizontal long axis was perpendicular to the light beam.

**Supplementary Tables with Legends**

**Supplementary Table S1.** Pattern, colour and name of the 8 smooth test surfaces composed of cardboard squares (15 cm × 15 cm) used for schlieren imaging (Supplementary Fig. S2).

| **smooth test surfaces** | | | |
| --- | --- | --- | --- |
| **pattern** | **colour** | **name** | **panel in Fig. S2** |
| homogeneous | black | shb | A |
| homogeneous | grey | shg | B |
| homogeneous | white | shw | C |
| striped | 7.5 cm stripe width  (1 black + 1 white stripes) | ss7.5(1b1w) | D |
| striped | 5 cm stripe width  (2 black + 1 white stripes) | ss5(2b1w) | E |
| striped | 3 cm stripe width  (3 black + 2 white stripes) | ss3(3b2w) | F |
| striped | 1 cm stripe width  (8 black + 7 white stripes) | ss1(8b7w) | G |
| striped | 0.5 cm stripe width  (15 black + 15 white stripes) | ss0.5(15b15w) | H |

**Supplementary Table S2.** Pattern, colour and name of the 10 hairy test surfaces composed of cattle, horse and zebra hides glued by dextrin to a cylindrical (length: 15 cm, radius: 7 cm) gypsum base used for schlieren imaging (Supplementary Fig. S3).

| **hairy test surfaces** | | | |
| --- | --- | --- | --- |
| **pattern** | **colour** | **name** | **panel in Fig. S3** |
| homogeneous | dark brown horse hide, short hair | hhbh | A |
| homogeneous | white cattle hide, short hair | hhwc | B |
| homogeneous | grey cattle hide, long hair | hhgc | C |
| homogeneous | black cattle hide, long hair | hhbc | D |
| striped | cattle hide, 1 cm stripe width  (7 long-haired black stripes +  7 short-haired white stripes  perpendicular to the cylinder’s long axis) | hsc1(7b7w)perp | E |
| striped | cattle hide, 1 cm stripe width  (8 long-haired black stripes +  7 short-haired white stripes  parallel to the cylinder’s long axis) | hsc1(8b7w)par | F |
| striped | cattle hide, 3 cm stripe width  (3 long-haired black stripes +  2 short-haired white stripes  perpendicular to the cylinder’s long axis) | hsc3(3b2w)perp | G |
| striped | horse hide, 1 cm stripe width  (8 short-haired dark brown stripes +  8 short-haired white stripes  perpendicular to the cylinder’s long axis) | hsh1(8b8w)perp | H |
| striped | zebra hide, front leg,  average white stripe width: 2 cm,  average black stripe width: 1 cm  (8 short-haired black stripes +  7 short-haired white stripes  perpendicular to the cylinder’s long axis) | hsz(8b7w)perp | I |
| striped | grey cattle hide, 3 cm stripe width  (3 short-haired stripes + 2 long-haired stripes  perpendicular to the cylinder’s long axis) | hsgc3(3s2L)perp | J |

**Supplementary Table S3.** Average surface temperature *T*_ave_ (^o^C), standard deviation σ_T_ (^o^C), minimum *T*_min_ (^o^C), maximum *T*_max_ (^o^C) and average temperature difference Δ*T* ± σ_ΔT_ (^o^C) between the adjacent local minima and maxima of *T* measured with thermography along the horizontal straight lines of lamplit smooth test surfaces in Fig. 3.

| **smooth test surface** | ***T*_ave_ (^o^C)** | **σ_T_ (^o^C)** | ***T*_min_ (^o^C)** | ***T*_max_ (^o^C)** | **Δ*T* (^o^C)** | **σ_ΔT_ (^o^C)** |
| --- | --- | --- | --- | --- | --- | --- |
| **shw** | 47.3 | 1.6 | 43.8 | 49.2 | - | - |
| **shg** | 50.4 | 1.7 | 46.6 | 52.4 | - | - |
| **ss0.5(15b15w)** | 60.0 | 2.9 | 53.5 | 65.0 | 4.3 | 0.6 |
| **ss7.5(1b1w)** | 62.5 | 12.2 | 46.1 | 77.5 | 27.2 | - |
| **ss1(8b7w)** | 63.7 | 5.3 | 53.5 | 73.3 | 12.9 | 0.9 |
| **ss3(3b2w)** | 65.5 | 9.6 | 52.3 | 81.6 | 16.2 | 12.1 |
| **ss5(2b1w)** | 65.5 | 9.6 | 51.1 | 75.3 | 23.2 | - |
| **shb** | 70.1 | 3.0 | 63.4 | 74.3 | - | - |

**Supplementary Table S4.** Average surface temperature *T*_ave_ (^o^C), standard deviation σ_T_ (^o^C), minimum *T*_min_ (^o^C), maximum *T*_max_ (^o^C) and average temperature difference Δ*T* ± σ_ΔT_ (^o^C) between the adjacent local minima and maxima of *T*_ave_ measured with thermography along the horizontal straight lines of lamplit hairy test surfaces in Fig. 4.

| **hairy test surface** | ***T*_ave_ (^o^C)** | **σ_T_ (^o^C)** | ***T*_min_ (^o^C)** | ***T*_max_ (^o^C)** | **Δ*T* (^o^C)** | **σ_ΔT_ (^o^C)** |
| --- | --- | --- | --- | --- | --- | --- |
| **hhwc** | 44.6 | 1.2 | 39.6 | 46.8 | 0.8 | 0.3 |
| **hsc1(8b7w)par** | 52.3 | 5.7 | 42.4 | 62.7 | 5.3 | 1.6 |
| **hsgc3(3s2L)perp** | 52.9 | 2.4 | 47.0 | 59.0 | 3.1 | 0.9 |
| **hhgc** | 53.7 | 2.7 | 45.8 | 58.5 | 3.8 | 1.9 |
| **hsz(8b7w)perp** | 55.2 | 1.5 | 49.7 | 57.9 | 1.5 | 0.9 |
| **hhbh** | 55.3 | 1.4 | 49.1 | 57.2 | 0.8 | 0.8 |
| **hsh1(8b8w)perp** | 56.4 | 1.7 | 51.3 | 59.4 | 1.7 | 0.8 |
| **hsc3(3b2w)perp** | 56.5 | 4.4 | 49.2 | 65.8 | 11.6 | 3.6 |
| **hhbc** | 63.2 | 3.8 | 51.9 | 69.4 | 2.7 | 1.8 |
| **hsc1(7b7w)perp** | 65.0 | 4.2 | 55.0 | 72.0 | 3.9 | 2.6 |

**Supplementary Table S5.** Results of the Wilcoxon rank-sum test applied for the smooth test surfaces. Because of the large sample size (4475 observations per surface) we randomly selected 250 observations from all surfaces, run the Wilcoxon rank-sum test, repeated it 499 times and averaged the results. The long axis of both homogeneous and striped cylindrical test surfaces was perpendicular and the curved stripes were parallel to the light beam. At the striped surfaces the stripes were perpendicular to the curvature. n.s.: p > 0.05, *: 0.001 <= p < 0.05, **: 0.0001 <= p < 0.001, ***: p < 0.0001.

| ***N*_min_(upper)** | ss0.5(15b15w) | ss1(8b7w) | ss3(3b2w) | ss5(2b1w) | ss7.5(1b1w) | shb | shg |
| --- | --- | --- | --- | --- | --- | --- | --- |
| ss1(8b7w) | n.s. | - | - | - | - | - | - |
| ss3(3b2w) | n.s. | n.s. | - | - | - | - | - |
| ss5(2b1w) | n.s. | n.s. | * | - | - | - | - |
| ss7.5(1b1w) | n.s. | n.s. | n.s. | n.s. | - | - | - |
| shb | * | ** | n.s. | *** | n.s. | - | - |
| shg | *** | *** | *** | * | *** | *** | - |
| shw | *** | *** | *** | *** | *** | *** | n.s. |
|  | | | | | | | |
| ***N*_min_(lower)** | ss0.5(15b15w) | ss1(8b7w) | ss3(3b2w) | ss5(2b1w) | ss7.5(1b1w) | shb | shg |
| ss1(8b7w) | *** | - | - | - | - | - | - |
| ss3(3b2w) | n.s. | *** | - | - | - | - | - |
| ss5(2b1w) | n.s. | *** | n.s. | - | - | - | - |
| ss7.5(1b1w) | * | *** | *** | n.s. | - | - | - |
| shb | n.s. | *** | n.s. | n.s. | * | - | - |
| shg | *** | *** | *** | *** | *** | *** | - |
| shw | *** | *** | *** | *** | *** | *** | * |
|  | | | | | | | |
| ***d*_ave_(upper)** | ss0.5(15b15w) | ss1(8b7w) | ss3(3b2w) | ss5(2b1w) | ss7.5(1b1w) | shb | shg |
| ss1(8b7w) | n.s. | - | - | - | - | - | - |
| ss3(3b2w) | n.s. | n.s. | - | - | - | - | - |
| ss5(2b1w) | n.s. | n.s. | * | - | - | - | - |
| ss7.5(1b1w) | n.s. | n.s. | n.s. | n.s. | - | - | - |
| shb | * | * | n.s. | *** | n.s. | - | - |
| shg | *** | *** | *** | * | *** | *** | - |
| shw | *** | *** | *** | *** | *** | *** | n.s. |
|  | | | | | | | |
| ***d*_ave_(lower)** | ss0.5(15b15w) | ss1(8b7w) | ss3(3b2w) | ss5(2b1w) | ss7.5(1b1w) | shb | shg |
| ss1(8b7w) | *** | - | - | - | - | - | - |
| ss3(3b2w) | n.s. | *** | - | - | - | - | - |
| ss5(2b1w) | n.s. | * | n.s. | - | - | - | - |
| ss7.5(1b1w) | n.s. | * | n.s. | n.s. | - | - | - |
| shb | n.s. | *** | n.s. | n.s. | n.s. | - | - |
| shg | *** | *** | *** | *** | *** | *** | - |
| shw | *** | *** | *** | *** | *** | *** | n.s. |
|  | | | | | | | |
| **Δ*I*(upper)** | ss0.5(15b15w) | ss1(8b7w) | ss3(3b2w) | ss5(2b1w) | ss7.5(1b1w) | shb | shg |
| ss1(8b7w) | n.s. | - | - | - | - | - | - |
| ss3(3b2w) | n.s. | n.s. | - | - | - | - | - |
| ss5(2b1w) | n.s. | n.s. | n.s. | - | - | - | - |
| ss7.5(1b1w) | n.s. | n.s. | n.s. | n.s. | - | - | - |
| shb | ** | ** | n.s. | *** | * | - | - |
| shg | *** | *** | *** | *** | *** | *** | - |
| shw | *** | *** | *** | *** | *** | *** | n.s. |
|  | | | | | | | |
| **Δ*I*(lower)** | ss0.5(15b15w) | ss1(8b7w) | ss3(3b2w) | ss5(2b1w) | ss7.5(1b1w) | shb | shg |
| ss1(8b7w) | *** | - | - | - | - | - | - |
| ss3(3b2w) | * | * | - | - | - | - | - |
| ss5(2b1w) | n.s. | *** | * | - | - | - | - |
| ss7.5(1b1w) | n.s. | *** | n.s. | n.s. | - | - | - |
| shb | n.s. | * | n.s. | n.s. | n.s. | - | - |
| shg | *** | *** | *** | *** | *** | *** | - |
| shw | *** | *** | *** | *** | *** | *** | * |

**Supplementary Table S6.** Variances explained by the principal components. The PCA analysis was applied to the smooth test surfaces, with the *N*_min_(upper), *d*_ave_(upper), Δ*I*(upper), *N*_min_(lower), *d*_ave_(lower) and Δ*I*(lower) variables. The long axis of both homogeneous and striped cylindrical test surfaces was perpendicular and the curved stripes were parallel to the light beam.

|  | **PC1** | **PC2** | **PC3** | **PC4** | **PC5** | **PC6** |
| --- | --- | --- | --- | --- | --- | --- |
| **standard deviation** | 1.6942 | 1.1542 | 0.7809 | 0.72586 | 0.6587 | 0.47631 |
| **proportion of variance** | 0.4784 | 0.222 | 0.1016 | 0.08781 | 0.07231 | 0.03781 |
| **cumulative proportion** | 0.4784 | 0.7004 | 0.8021 | 0.88987 | 0.96219 | 1 |

**Supplementary Table S7.** Results of the Wilcoxon rank-sum test applied for the air stream behaviour variables of smooth test surfaces. The long axis of both homogeneous and striped cylindrical test surfaces was perpendicular and the curved stripes were parallel to the light beam. Only air streams with lifespan *t* > 1 second were considered. n.s.: p > 0.05, *: 0.001 <= p < 0.05, **: 0.0001 <= p < 0.001, ***: p < 0.0001.

| **lifespan *t*** | ss0.5(15b15w) | ss1(8b7w) | ss3(3b2w) | ss5(2b1w) | ss7.5(1b1w) | shb | shg |
| --- | --- | --- | --- | --- | --- | --- | --- |
| ss1(8b7w) | *** | - | - | - | - | - | - |
| ss3(3b2w) | * | n.s. | - | - | - | - | - |
| ss5(2b1w) | n.s. | * | n.s. | - | - | - | - |
| ss7.5(1b1w) | n.s. | * | n.s. | n.s. | - | - | - |
| shb | n.s. | *** | * | n.s. | n.s. | - | - |
| shg | n.s. | * | n.s. | n.s. | n.s. | n.s. | - |
| shw | n.s. | *** | * | n.s. | n.s. | n.s. | n.s. |
|  | | | | | | | |
| **covered**  **distance**  ***d*_covered_** | ss0.5(15b15w) | ss1(8b7w) | ss3(3b2w) | ss5(2b1w) | ss7.5(1b1w) | shb | shg |
| ss1(8b7w) | n.s. | - | - | - | - | - | - |
| ss3(3b2w) | n.s. | n.s. | - | - | - | - | - |
| ss5(2b1w) | * | * | n.s. | - | - | - | - |
| ss7.5(1b1w) | * | ** | * | n.s. | - | - | - |
| shb | * | *** | * | n.s. | n.s. | - | - |
| shg | n.s. | n.s. | n.s. | n.s. | n.s. | n.s. | - |
| shw | n.s. | n.s. | n.s. | n.s. | n.s. | n.s. | n.s. |
|  | | | | | | | |
| **mean speed**  ***v*_mean_** | ss0.5(15b15w) | ss1(8b7w) | ss3(3b2w) | ss5(2b1w) | ss7.5(1b1w) | shb | shg |
| ss1(8b7w) | * | - | - | - | - | - | - |
| ss3(3b2w) | n.s. | * | - | - | - | - | - |
| ss5(2b1w) | n.s. | *** | * | - | - | - | - |
| ss7.5(1b1w) | n.s. | *** | * | n.s. | - | - | - |
| shb | * | n.s. | *** | n.s. | n.s. | - | - |
| shg | n.s. | *** | * | n.s. | n.s. | n.s. | - |
| shw | n.s. | *** | n.s. | n.s. | n.s. | n.s. | n.s. |
|  | | | | | | | |
| **maximum**  **distance *d_max_*** | ss0.5(15b15w) | ss1(8b7w) | ss3(3b2w) | ss5(2b1w) | ss7.5(1b1w) | shb | shg |
| ss1(8b7w) | n.s. | - | - | - | - | - | - |
| ss3(3b2w) | n.s. | n.s. | - | - | - | - | - |
| ss5(2b1w) | n.s. | *** | n.s. | - | - | - | - |
| ss7.5(1b1w) | * | *** | * | n.s. | - | - | - |
| shb | * | *** | * | n.s. | n.s. | - | - |
| shg | n.s. | ** | n.s. | n.s. | n.s. | n.s. | - |
| shw | n.s. | * | n.s. | n.s. | n.s. | n.s. | n.s. |
|  | | | | | | | |
| **start-end**  **distance *d*_se_** | ss0.5(15b15w) | ss1(8b7w) | ss3(3b2w) | ss5(2b1w) | ss7.5(1b1w) | shb | shg |
| ss1(8b7w) | n.s. | - | - | - | - | - | - |
| ss3(3b2w) | n.s. | n.s. | - | - | - | - | - |
| ss5(2b1w) | n.s. | * | n.s. | - | - | - | - |
| ss7.5(1b1w) | n.s. | ** | * | n.s. | - | - | - |
| shb | n.s. | *** | * | n.s. | n.s. | - | - |
| shg | n.s. | ** | * | n.s. | n.s. | n.s. | - |
| shw | n.s. | * | n.s. | n.s. | n.s. | n.s. | n.s. |

**Supplementary Table S8.** Variances explained by the principal components. The PCA analysis was applied to the smooth test surfaces, with the lifespan *t*, covered distance *d*_covered_, mean speed *v*_mean_, maximum distance *d*_max_ and start-end distance *d*_se_ variables. The long axis of both homogeneous and striped cylindrical test surfaces was perpendicular and the curved stripes were parallel to the light beam. Only air streams with lifespan *t* > 1 second were considered.

|  | **PC1** | **PC2** | **PC3** | **PC4** | **PC5** |
| --- | --- | --- | --- | --- | --- |
| **standard deviation** | 1.897 | 1.0828 | 0.37508 | 0.26001 | 0.14447 |
| **proportion of variance** | 0.7197 | 0.2345 | 0.02814 | 0.01352 | 0.00417 |
| **cumulative proportion** | 0.7197 | 0.9542 | 0.98231 | 0.99583 | 1 |

**Supplementary Table S9.** Results of the Wilcoxon rank-sum test applied for the hairy test surfaces. Because of the large sample size (5369 observations per surface) we randomly selected 250 observations from all surfaces, run the Wilcoxon rank-sum test, repeated it 499 times and averaged the results. The long axis of the cylindrical striped test surfaces was perpendicular and the curved stripes were parallel to the light beam for hsz(8b7w)perp, hsh1(8b8w)perp, hsc1(7b7w)perp, hsgc3(3s2L)perp, hsc3(3b2w)perp. For hsc1(8b7w)par the horizontal stripes were parallel and perpendicular to the light beam. For hhwc, hhgc, hhbh and hhbc the long axis of the cylindrical homogeneous test surfaces was perpendicular to the light beam. n.s.: p > 0.05, *: 0.001 <= p < 0.05, **: 0.0001 <= p < 0.001, ***: p < 0.0001.

| ***N*_min_(upper)** | hsz  (8b7w)  perp | hsh1  (8b8w)  perp | hsc1  (7b7w)  perp | hsc1  (8b7w)  par  normal  to beam | hsc1  (8b7w)  par  parallel  to beam | hsgc3  (3s2L)  perp | hsc3  (3b2w)  perp | hhwc | hhgc | hhbh |
| --- | --- | --- | --- | --- | --- | --- | --- | --- | --- | --- |
| hsh1(8b8w)  perp | n.s. | - | - | - | - | - | - | - | - | - |
| hsc1(7b7w)  perp | *** | *** | - | - | - | - | - | - | - | - |
| hsc1(8b7w)  par  normal  to beam | *** | *** | n.s. | - | - | - | - | - | - | - |
| hsc1(8b7w)  par  parallel  to beam | *** | *** | *** | *** | - | - | - | - | - | - |
| hsgc3(3s2L)  perp | *** | *** | * | n.s. | *** | - | - | - | - | - |
| hsc3(3b2w)  perp | *** | *** | n.s. | n.s. | *** | * | - | - | - | - |
| hhwc | *** | n.s. | *** | *** | *** | *** | *** | - | - | - |
| hhgc | *** | *** | *** | *** | *** | ** | *** | *** | - | - |
| hhbh | n.s. | n.s. | *** | *** | *** | *** | *** | * | *** | - |
| hhbc | *** | *** | n.s. | n.s. | *** | *** | n.s. | *** | *** | *** |
|  | | | | | | | | | | |
| ***N*_min_(lower)** | hsz  (8b7w)  perp | hsh1  (8b8w)  perp | hsc1  (7b7w)  perp | hsc1  (8b7w)  par  normal  to beam | hsc1  (8b7w)  par  parallel  to beam | hsgc3  (3s2L)  perp | hsc3  (3b2w)  perp | hhwc | hhgc | hhbh |
| hsh1(8b8w)  perp | *** | - | - | - | - | - | - | - | - | - |
| hsc1(7b7w)  perp | *** | *** | - | - | - | - | - | - | - | - |
| hsc1(8b7w)  par  normal  to beam | *** | *** | *** | - | - | - | - | - | - | - |
| hsc1(8b7w)  par  parallel  to beam | *** | ** | *** | n.s. | - | - | - | - | - | - |
| hsgc3(3s2L)  perp | *** | n.s. | *** | *** | * | - | - | - | - | - |
| hsc3(3b2w)  perp | *** | *** | *** | n.s. | n.s. | *** | - | - | - | - |
| hhwc | *** | *** | *** | *** | *** | *** | *** | - | - | - |
| hhgc | *** | *** | *** | n.s. | n.s. | *** | n.s. | *** | - | - |
| hhbh | n.s. | *** | *** | *** | *** | *** | *** | *** | *** | - |
| hhbc | *** | *** | n.s. | *** | *** | *** | *** | *** | *** | *** |
|  | | | | | | | | | | |
| ***d*_ave_(upper)** | hsz  (8b7w)  perp | hsh1  (8b8w)  perp | hsc1  (7b7w)  perp | hsc1  (8b7w)  par  normal  to beam | hsc1  (8b7w)  par  parallel  to beam | hsgc3  (3s2L)  perp | hsc3  (3b2w)  perp | hhwc | hhgc | hhbh |
| hsh1(8b8w)  perp | n.s. | - | - | - | - | - | - | - | - | - |
| hsc1(7b7w)  perp | *** | *** | - | - | - | - | - | - | - | - |
| hsc1(8b7w)  par  normal  to beam | *** | *** | n.s. | - | - | - | - | - | - | - |
| hsc1(8b7w)  par  parallel  to beam | *** | *** | *** | *** | - | - | - | - | - | - |
| hsgc3(3s2L)  perp | *** | *** | n.s. | n.s. | *** | - | - | - | - | - |
| hsc3(3b2w)  perp | *** | *** | n.s. | n.s. | *** | n.s. | - | - | - | - |
| hhwc | * | n.s. | *** | *** | *** | *** | *** | - | - | - |
| hhgc | *** | *** | *** | *** | *** | * | *** | *** | - | - |
| hhbh | n.s. | n.s. | *** | *** | *** | *** | *** | n.s. | *** | - |
| hhbc | *** | *** | n.s. | n.s. | *** | n.s. | n.s. | *** | *** | *** |
|  | | | | | | | | | | |
| ***d*_ave_(lower)** | hsz  (8b7w)  perp | hsh1  (8b8w)  perp | hsc1  (7b7w)  perp | hsc1  (8b7w)  par  normal  to beam | hsc1  (8b7w)  par  parallel  to beam | hsgc3  (3s2L)  perp | hsc3  (3b2w)  perp | hhwc | hhgc | hhbh |
| hsh1(8b8w)  perp | * | - | - | - | - | - | - | - | - | - |
| hsc1(7b7w)  perp | *** | *** | - | - | - | - | - | - | - | - |
| hsc1(8b7w)  par  normal  to beam | *** | *** | *** | - | - | - | - | - | - | - |
| hsc1(8b7w)  par  parallel  to beam | *** | *** | * | * | - | - | - | - | - | - |
| hsgc3(3s2L)  perp | *** | *** | *** | n.s. | n.s. | - | - | - | - | - |
| hsc3(3b2w)  perp | *** | *** | *** | n.s. | * | n.s. | - | - | - | - |
| hhwc | *** | *** | *** | *** | *** | *** | *** | - | - | - |
| hhgc | *** | *** | *** | n.s. | * | * | n.s. | *** | - | - |
| hhbh | n.s. | *** | *** | *** | *** | *** | *** | * | *** | - |
| hhbc | *** | *** | * | *** | n.s. | n.s. | *** | *** | *** | *** |
|  | | | | | | | | | | |
| **Δ*I*(upper)** | hsz  (8b7w)  perp | hsh1  (8b8w)  perp | hsc1  (7b7w)  perp | hsc1  (8b7w)  par  normal  to beam | hsc1  (8b7w)  par  parallel  to beam | hsgc3  (3s2L)  perp | hsc3  (3b2w)  perp | hhwc | hhgc | hhbh |
| hsh1(8b8w)  perp | n.s. | - | - | - | - | - | - | - | - | - |
| hsc1(7b7w)  perp | *** | *** | - | - | - | - | - | - | - | - |
| hsc1(8b7w)  par  normal  to beam | *** | *** | n.s. | - | - | - | - | - | - | - |
| hsc1(8b7w)  par  parallel  to beam | *** | *** | *** | *** | - | - | - | - | - | - |
| hsgc3(3s2L)  perp | *** | *** | * | n.s. | *** | - | - | - | - | - |
| hsc3(3b2w)  perp | *** | *** | n.s. | * | *** | *** | - | - | - | - |
| hhwc | *** | *** | *** | *** | *** | *** | *** | - | - | - |
| hhgc | *** | *** | *** | *** | *** | *** | *** | *** | - | - |
| hhbh | n.s. | n.s. | *** | *** | *** | *** | *** | *** | *** | - |
| hhbc | *** | *** | * | ** | *** | *** | n.s. | *** | *** | *** |
|  | | | | | | | | | | |
| **Δ*I*(lower)** | hsz  (8b7w)  perp | hsh1  (8b8w)  perp | hsc1  (7b7w)  perp | hsc1  (8b7w)  par  normal  to beam | hsc1  (8b7w)  par  parallel  to beam | hsgc3  (3s2L)  perp | hsc3  (3b2w)  perp | hhwc | hhgc | hhbh |
| hsh1(8b8w)  perp | *** | - | - | - | - | - | - | - | - | - |
| hsc1(7b7w)  perp | *** | *** | - | - | - | - | - | - | - | - |
| hsc1(8b7w)  par  normal  to beam | *** | *** | *** | - | - | - | - | - | - | - |
| hsc1(8b7w)  par  parallel  to beam | *** | *** | n.s. | *** | - | - | - | - | - | - |
| hsgc3(3s2L)  perp | *** | *** | *** | *** | *** | - | - | - | - | - |
| hsc3(3b2w)  perp | *** | *** | n.s. | *** | n.s. | *** | - | - | - | - |
| hhwc | *** | *** | *** | *** | *** | *** | *** | - | - | - |
| hhgc | *** | n.s. | *** | *** | *** | *** | *** | *** | - | - |
| hhbh | *** | *** | *** | *** | *** | *** | *** | *** | *** | - |
| hhbc | *** | *** | *** | * | *** | n.s. | *** | *** | *** | *** |

**Supplementary Table S10.** Variances explained by the principal components. The PCA analysis was applied to the hairy test surfaces, with the *N*_min_(upper), *d*_ave_(upper), Δ*I*(upper), *N*_min_(lower), *d*_ave_(lower) and Δ*I*(lower) variables. The long axis of the cylindrical striped test surfaces was perpendicular and the curved stripes were parallel to the light beam for hsz(8b7w)perp, hsh1(8b8w)perp, hsc1(7b7w)perp, hsgc3(3s2L)perp, hsc3(3b2w)perp. For hsc1(8b7w)par the horizontal stripes were parallel and perpendicular to the light beam. For hhwc, hhgc, hhbh and hhbc the long axis of the cylindrical homogeneous test surfaces was perpendicular to the light beam.

|  | **PC1** | **PC2** | **PC3** | **PC4** | **PC5** | **PC6** |
| --- | --- | --- | --- | --- | --- | --- |
| **standard deviation** | 1.9593 | 0.9202 | 0.6587 | 0.602 | 0.55149 | 0.46231 |
| **proportion of variance** | 0.6398 | 0.1411 | 0.07231 | 0.0604 | 0.05069 | 0.03562 |
| **cumulative proportion** | 0.6398 | 0.781 | 0.85329 | 0.9137 | 0.96438 | 1 |

**Supplementary Table S11.** Results of the Wilcoxon rank-sum test applied for the hairy test surfaces. The long axis of the cylindrical striped test surfaces was perpendicular and the curved stripes were parallel to the light beam for hsz(8b7w)perp, hsh1(8b8w)perp, hsc1(7b7w)perp, hsgc3(3s2L)perp, hsc3(3b2w)perp. For hsc1(8b7w)par the horizontal stripes were parallel and perpendicular to the light beam. For hhwc, hhgc, hhbh and hhbc the long axis of the cylindrical homogeneous test surfaces was perpendicular to the light beam. Only air streams with lifespan *t* > 1 second were considered. n.s.: p > 0.05, *: 0.001 <= p < 0.05, **: 0.0001 <= p < 0.001, ***: p < 0.0001.

| **lifespan *t*** | hsz  (8b7w)  perp | hsh1  (8b8w)  perp | hsc1  (7b7w)  perp | hsc1  (8b7w)  par  normal  to beam | hsc1  (8b7w)  par  parallel  to beam | hsgc3  (3s2L)  perp | hsc3  (3b2w)  perp | hhwc | hhgc | hhbh |
| --- | --- | --- | --- | --- | --- | --- | --- | --- | --- | --- |
| hsh1(8b8w)  perp | *** | - | - | - | - | - | - | - | - | - |
| hsc1(7b7w)  perp | *** | n.s. | - | - | - | - | - | - | - | - |
| hsc1(8b7w)  par  normal  to beam | n.s. | *** | *** | - | - | - | - | - | - | - |
| hsc1(8b7w)  par  parallel  to beam | n.s. | *** | *** | n.s. | - | - | - | - | - | - |
| hsgc3(3s2L)  perp | n.s. | * | ** | n.s. | n.s. | - | - | - | - | - |
| hsc3(3b2w)  perp | n.s. | n.s. | n.s. | n.s. | n.s. | n.s. | - | - | - | - |
| hhwc | n.s. | *** | *** | *** | * | ** | *** | - | - | - |
| hhgc | n.s. | * | ** | n.s. | n.s. | n.s. | n.s. | *** | - | - |
| hhbh | n.s. | *** | *** | ** | n.s. | ** | ** | n.s. | *** | - |
| hhbc | n.s. | ** | *** | n.s. | n.s. | n.s. | n.s. | *** | n.s. | *** |
|  | | | | | | | | | | |
| **covered**  **distance**  ***d*_covered_** | hsz  (8b7w)  perp | hsh1  (8b8w)  perp | hsc1  (7b7w)  perp | hsc1  (8b7w)  par  normal  to beam | hsc1  (8b7w)  par  parallel  to beam | hsgc3  (3s2L)  perp | hsc3  (3b2w)  perp | hhwc | hhgc | hhbh |
| hsh1(8b8w)  perp | n.s. | - | - | - | - | - | - | - | - | - |
| hsc1(7b7w)  perp | n.s. | n.s. | - | - | - | - | - | - | - | - |
| hsc1(8b7w)  par  normal  to beam | * | *** | *** | - | - | - | - | - | - | - |
| hsc1(8b7w)  par  parallel  to beam | * | *** | *** | n.s. | - | - | - | - | - | - |
| hsgc3(3s2L)  perp | n.s. | n.s. | n.s. | ** | *** | - | - | - | - | - |
| hsc3(3b2w)  perp | * | n.s. | n.s. | *** | *** | * | - | - | - | - |
| hhwc | n.s. | * | n.s. | n.s. | n.s. | n.s. | ** | - | - | - |
| hhgc | n.s. | n.s. | n.s. | *** | *** | n.s. | n.s. | * | - | - |
| hhbh | * | *** | *** | n.s. | n.s. | *** | *** | n.s. | *** | - |
| hhbc | n.s. | * | n.s. | * | * | n.s. | *** | n.s. | * | * |
|  | | | | | | | | | | |
| **mean**  **speed**  ***v*_mean_** | hsz  (8b7w)  perp | hsh1  (8b8w)  perp | hsc1  (7b7w)  perp | hsc1  (8b7w)  par  normal  to beam | hsc1  (8b7w)  par  parallel  to beam | hsgc3  (3s2L)  perp | hsc3  (3b2w)  perp | hhwc | hhgc | hhbh |
| hsh1(8b8w)  perp | *** | - | - | - | - | - | - | - | - | - |
| hsc1(7b7w)  perp | *** | n.s. | - | - | - | - | - | - | - | - |
| hsc1(8b7w)  par  normal  to beam | * | n.s. | n.s. | - | - | - | - | - | - | - |
| hsc1(8b7w)  par  parallel  to beam | * | n.s. | n.s. | n.s. | - | - | - | - | - | - |
| hsgc3(3s2L)  perp | n.s. | *** | *** | *** | *** | - | - | - | - | - |
| hsc3(3b2w)  perp | *** | n.s. | n.s. | n.s. | n.s. | *** | - | - | - | - |
| hhwc | * | n.s. | n.s. | n.s. | n.s. | *** | n.s. | - | - | - |
| hhgc | *** | ** | n.s. | n.s. | n.s. | n.s. | *** | *** | - | - |
| hhbh | *** | n.s. | n.s. | *** | ** | *** | n.s. | n.s. | n.s. | - |
| hhbc | n.s. | n.s. | *** | *** | *** | n.s. | n.s. | *** | *** | *** |
|  | | | | | | | | | | |
| **maximum**  **distance**  ***d*_max_** | hsz  (8b7w)  perp | hsh1  (8b8w)  perp | hsc1  (7b7w)  perp | hsc1  (8b7w)  par  normal  to beam | hsc1  (8b7w)  par  parallel  to beam | hsgc3  (3s2L)  perp | hsc3  (3b2w)  perp | hhwc | hhgc | hhbh |
| hsh1(8b8w)  perp | * | - | - | - | - | - | - | - | - | - |
| hsc1(7b7w)  perp | n.s. | n.s. | - | - | - | - | - | - | - | - |
| hsc1(8b7w)  par  normal  to beam | ** | n.s. | *** | - | - | - | - | - | - | - |
| hsc1(8b7w)  par  parallel  to beam | * | *** | *** | n.s. | - | - | - | - | - | - |
| hsgc3(3s2L)  perp | n.s. | n.s. | n.s. | *** | *** | - | - | - | - | - |
| hsc3(3b2w)  perp | *** | n.s. | *** | n.s. | n.s. | *** | - | - | - | - |
| hhwc | n.s. | *** | n.s. | n.s. | n.s. | n.s. | *** | - | - | - |
| hhgc | n.s. | n.s. | n.s. | *** | *** | n.s. | ** | * | - | - |
| hhbh | *** | *** | *** | n.s. | n.s. | *** | n.s. | n.s. | *** | - |
| hhbc | n.s. | *** | n.s. | ** | n.s. | n.s. | *** | n.s. | ** | *** |
|  | | | | | | | | | | |
| **start-end**  **distance *d*_se_** | hsz  (8b7w)  perp | hsh1  (8b8w)  perp | hsc1  (7b7w)  perp | hsc1  (8b7w)  par  normal  to beam | hsc1  (8b7w)  par  parallel  to beam | hsgc3  (3s2L)  perp | hsc3  (3b2w)  perp | hhwc | hhgc | hhbh |
| hsh1(8b8w)  perp | * | - | - | - | - | - | - | - | - | - |
| hsc1(7b7w)  perp | n.s. | n.s. | - | - | - | - | - | - | - | - |
| hsc1(8b7w)  par  normal  to beam | n.s. | *** | *** | - | - | - | - | - | - | - |
| hsc1(8b7w)  par  parallel  to beam | n.s. | *** | *** | n.s. | - | - | - | - | - | - |
| hsgc3(3s2L)  perp | n.s. | n.s. | n.s. | *** | *** | - | - | - | - | - |
| hsc3(3b2w)  perp | *** | n.s. | * | n.s. | n.s. | *** | - | - | - | - |
| hhwc | n.s. | *** | ** | n.s. | n.s. | * | *** | - | - | - |
| hhgc | n.s. | n.s. | n.s. | *** | *** | n.s. | * | ** | - | - |
| hhbh | ** | *** | *** | n.s. | n.s. | *** | *** | n.s. | *** | - |
| hhbc | n.s. | *** | n.s. | * | * | n.s. | *** | n.s. | * | *** |

**Supplementary Table S12.** Variances explained by the principal components. The PCA analysis was applied to the hairy test surfaces, with the lifespan *t*, covered distance *d*_covered_, mean speed *v*_mean_, maximum distance *d*_max_ and start-end distance *d*_se_ variables. The long axis of the cylindrical striped test surfaces was perpendicular and the curved stripes were parallel to the light beam for hsz(8b7w)perp, hsh1(8b8w)perp, hsc1(7b7w)perp, hsgc3(3s2L)perp, hsc3(3b2w)perp. For hsc1(8b7w)par the horizontal stripes were parallel and perpendicular to the light beam. For hhwc, hhgc, hhbh and hhbc the long axis of the cylindrical homogeneous test surfaces was perpendicular to the light beam. Only air streams with lifespan *t* > 1 second were considered.

|  | **PC1** | **PC2** | **PC3** | **PC4** | **PC5** |
| --- | --- | --- | --- | --- | --- |
| **standard deviation** | 1.8085 | 1.1697 | 0.47792 | 0.30787 | 0.19521 |
| **proportion of variance** | 0.6541 | 0.2736 | 0.04568 | 0.01896 | 0.00762 |
| **cumulative proportion** | 0.6541 | 0.9277 | 0.97342 | 0.99238 | 1 |
